# Full-length transcriptome atlas of gallbladder cancer reveals trastuzumab resistance conferred by ERBB2 alternative splicing

**DOI:** 10.1038/s41392-025-02150-w

**Published:** 2025-02-14

**Authors:** Ziyi Wang, Li Gao, Ziheng Jia, Liguo Liu, Ao Gu, Zhaonan Liu, Qin Zhu, Yichen Zuo, Mingjie Yang, Shijia Wang, Jiyao Ma, Jingyun Zhang, Shimei Qiu, Zhizhen Li, Jinghan Wang, Dongxi Xiang, Fatao Liu, Rong Shao, Yanjing Li, Maolan Li, Wu Wei, Yingbin Liu

**Affiliations:** 1 Department of Biliary-Pancreatic Surgery, Ren Ji Hospital, Shanghai Jiao Tong University School of Medicine, Shanghai, China; 2 Shanghai Key Laboratory for Cancer Systems Regulation and Clinical Translation (CSRCT-SHANGHAI), Renji Hospital Affiliated to Shanghai Jiao Tong University School of Medicine, Shanghai, China; 3 State Key Laboratory of Systems Medicine for Cancer, Shanghai Cancer Institute, Renji Hospital Affiliated to Shanghai Jiao Tong University school of Medicine, Shanghai, China; 4 CAS Key Laboratory of Computational Biology, Shanghai Institute of Nutrition and Health, University of Chinese Academy of Sciences, Chinese Academy of Sciences, Shanghai, China; 5 Changzhou No.2 People Hospital Affiliated to Nanjing Medical University, Changzhou, China; 6 Department of Biliary Tract Surgery I, Eastern Hepatobiliary Surgery Hospital, Shanghai, China; 7 Department of Hepatobiliary and Pancreatic Surgery, Institute of Hepatobiliary and Pancreatic Surgery, Shanghai East Hospital, School of Medicine, Tongji University, Shanghai, China; 8 Shanghai Key Laboratory of Biliary Tract Disease Research, Xinhua Hospital, Shanghai Jiao Tong University School of Medicine, Shanghai, China; 9 Department of Pharmacology and Biochemistry, Shanghai Jiao Tong University School of Medicine, Shanghai, China; 10 Lingang Laboratory, Shanghai, China; 11 Department of General Surgery, Jiading Branch of Renji Hospital Affiliated to Shanghai Jiao Tong University School of Medicine, Shanghai, China

**Keywords:** Genetics research, Cancer genetics, Gastrointestinal cancer

## Abstract

Aberrant RNA alternative splicing in cancer generates varied novel isoforms and protein variants that facilitate cancer progression. Here, we employed the advanced long-read full-length transcriptome sequencing on gallbladder normal tissues, tumors, and cell lines to establish a comprehensive full-length gallbladder transcriptomic atlas. It is of note that receptor tyrosine kinases were one of the most dynamic components with highly variable transcript, with Erb-B2 receptor tyrosine kinase 2 (ERBB2) as a prime representative. A novel transcript, designated ERBB2 i14e, was identified for encoding a novel functional protein, and its protein expression was elevated in gallbladder cancer and strongly associated with worse prognosis. With the regulation of splicing factors ESRP1/2, ERBB2 i14e was alternatively spliced from intron 14 and the encoded i14e peptide was proved to facilitate the interaction with ERBB3 and downstream signaling activation of AKT. ERBB2 i14e was inducible and its expression attenuated anti-ERBB2 treatment efficacy in tumor xenografts. Further studies with patient derived xenografts models validated that ERBB2 i14e blockage with antisense oligonucleotide enhanced the tumor sensitivity to trastuzumab and its drug conjugates. Overall, this study provides a gallbladder specific long-read transcriptome profile and discovers a novel mechanism of trastuzumab resistance, thus ultimately devising strategies to improve trastuzumab therapy.

## Introduction

Gallbladder cancer (GBC) is ranked as one of the most malignancies in the digestive system.^1,2^ The lack of early typical symptoms frequently leads to a delayed diagnosis, resulting in a large population of patients that miss the opportunity for surgery. Consequently, many GBC patients are limited to non-invasive treatment options such as chemotherapy and targeted therapy.^3^ Unfortunately, the paucity of basic research on GBC has resulted in a dearth of effective targeted therapeutic strategies. Over the past decades, receptor tyrosine kinases (RTKs) have emerged as a predominant membrane factor to deliver oncogenic cues into intracellular signaling activation.^4–7^ A number of laboratories including ours have demonstrated that ERBB2 possesses the strong ability to drive tumor development in cancers including breast cancer, ovarian cancer, esophageal cancer, gastric cancer and GBC.^8,9^ Accumulated genomic studies have reported that mutations in the ErbB pathway are genomic hallmarks for GBC, contributing to tumor growth, metastasis, and immune escape.^10–13^ Consequently, ERBB2 merges as the most promising therapeutic target for this poorly understood malignancy.^14–17^ Trastuzumab, targeting ERBB2, is the first monoclonal antibody approved by FDA for cancer therapy. Strikingly, approximate two-thirds of breast cancer patients were tolerant and developed resistance during the treatment.^18–20^ The unresponsiveness is attributed, at least in part, to the divergent forms of ERBB2 including mutations^21^ and abnormal dimerization.^22,23^ To date, antibody-drug conjugates (ADCs) targeting ERBB2 have made great progression and demonstrated promising therapeutic potential in various cancers.^24–26^ This has once again solidified ERBB2 as a highly attractive target for cancer therapy. However, the resistance to ERBB2-targeting therapy in GBC remains poorly understood as ERBB2 mutation acts as a primary factor to drive the malignancy of GBC.

RNA, transcribed from the nuclear genome, could be translated into proteins in cytosol, thus carrying multidimensional biological information. RNA alternative splicing, induced by various factors, generates mRNAs with different exon combinations, resulting in diverse protein isoforms from a single gene and regulating fundamental cellular processes. In tumors, in addition to genomic instability, altered tumor-specific splicing processes have been shown to promote various hallmarks of tumor progression.^27,28^ The aberrant splicing factors, RNA editing, and chromatin remodeling have been proved to regulate alternative splicing in cancers and profoundly influence tumorigenesis, DNA stability, proliferation, and apoptosis. Therefore, comprehensive full-length transcriptome atlas construction is a highly effective approach to investigate RNA alterations in tumor biology.

However, identification of individual splicing events largely depends on the detection techniques. The prevalent short-read sequencing at present can detect the existence of alternative splicing events and provide useful but limited information of highly complicated mixture of transcripts for one gene.^29^ Although this approach can inform the aberrant details, it fails to present the combination of those abnormalities in one whole transcript. Given the lack of comprehensive transcriptome profiles for certain cancer types, the long-read sequencing technology, also referred to third-generation sequencing or single-molecule sequencing could offer an advanced platform capable of precise identification of long and intact transcripts.^30,31^ For example, the Pacific Biosciences Circular Consensus Sequencing (CCS) mode provides significant potential, especially for transcriptome profiling. Within this mode, RNA is reverse transcribed into cDNA and subsequently converted into a single-stranded circular DNA molecule that undergoes iterative rounds of rolling circle sequencing within zero-mode waveguides.^32^ Importantly, each round of circular sequencing occurs on the original template, thereby avoiding the accumulation of sequencing errors that happened in PCR amplification based sequencing techniques. As a result, highly accurate consensus reads, termed Hi-Fi reads, can be generated with a precision of up to 99.99%. Furthermore, these reads can accurately span up to 20 kb, often covering the full length of nearly all known transcripts. Thus, the innovation with high efficiency has emerged as a powerful tool to study the pathological activity of alternative splicing, especially for long novel transcripts that play significant roles in carcinogenesis, development and drug resistance.

In this study, we performed the high-depth long-read transcriptomic sequencing on GBC and non-cancerous gallbladder from tissues together with cell lines to establish the GBC transcriptomic atlas that illustrates abundant novel transcript isoforms and genes. Based on the atlas, we discovered one new isoform of ERBB2 termed as ERBB2 i14e, which harbors an extra novel exon derived from intron 14. It could be detected in about one fifth of GBC samples and associated with worse prognosis. The extra ERBB2 il4e exon encodes 34 amino acid residues located in the ERBB2 extracellular IV domain, displaying strong ability to activate intracellular AKT pathway and promote cell proliferation. Interestingly, trastuzumab failed to bind with the novel ERBB2 due to the steric hindrance mediated by additional i14e peptide. Interfering the alternative splicing of ERBB2 i14e by antisense oligonucleotides could alleviate the acquired trastuzumab resistance. Overall, our study established a new paradigm for tumor research utilizing long-read transcriptome sequencing and constructed a full-length transcriptome atlas for gallbladder research. To the end, we demonstrated a novel ERBB2 isoform as a tumor-promoting factor in the development of GBC and deciphered a novel resistance mechanism of anti-ERBB2 treatment.

## Long-read full-length transcriptomic atlas reveals high diversity of receptor tyrosine kinases in gallbladder cancer

**Fig. 1:**
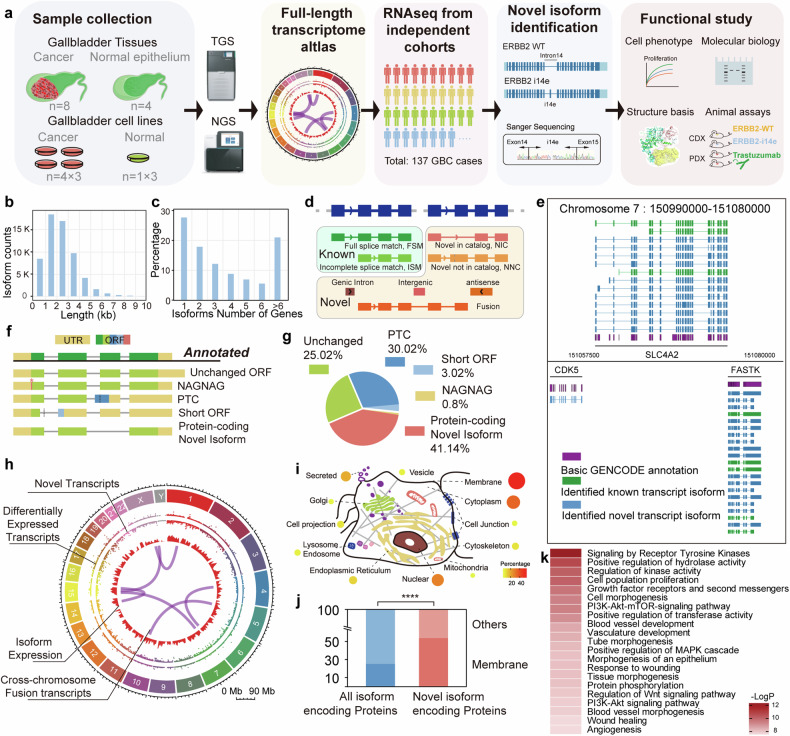
Establishment of gallbladder-gallbladder cancer transcriptome atlas. **a** Schematic representation of the research workflow. Transcriptomic atlas of normal gallbladder and gallbladder cancer together with gallbladder cell lines were constructed through sample collection, followed by the second and third-generation sequencing. Datasets of gallbladder cancer transcriptomes were engaged to identify novel transcripts followed by functional and molecular mechanistic studies. **b**, **c** Length distribution of detected transcripts and expression profiles of genes on transcript number were displayed. **d** Re-annotation of transcripts from *sqanti3* annotation into “known” and “novel” categories. **e** Regional landscape of full-length transcriptome in chromosome 7 involving CDK5, SLA4A2 and FASTK. **f** Five types of transcripts classification based on ORF property by *Transuit*. ORF open reading frame, PTC premature codon; **g** Distribution of these five classes among differentially expressed transcripts. **h** Transcriptomic landscape across chromosomes was displayed, in which inner arcs represented detected inter-chromosomal fusion transcripts. **i**, **j** Distribution of protein coding novel isoforms across all cellular compartments. Chi-square test was performed. **k** Gene Ontology analysis of top-ranked pathways associated with gallbladder cancer tumorigenesis and development

To comprehensively investigate the full-length transcriptomic signature of the GBC, we performed long-read transcriptome sequencing using PacBio Sequel II system on samples obtained from four normal gallbladder epitheliums, eight cases of GBC, four GBC cell lines, and one immortalized gallbladder epithelial cell line (L-2F7)^33^ (Fig. 1a). The median of sequencing depth was 1.28 TB, exceeding the saturation amount with 0.5 TB for known transcripts (Supplementary Fig. 1a). A total of 61,676 transcripts were detected from 13,109 genes in which 86.8% transcripts were from 1 kb to 4 kb in length, with a median of 3132 bp and the longest one detected was 13 kb of DYNC1H1 (Fig. 1b). 27.7% of genes expressed only one transcript form, while 21.2% expressed more than six isoforms, consistent with the previous long-read sequencing studies^34^ (Fig. 1c). The 5’ and 3’ end of the transcripts were examined with previous 5’ CAGE datasets and annotated polyadenylation sites (Supplementary Fig. 1b–e). The general read density was mapped (Supplementary Fig. 1f) and confirmed that the assembled long reads transcripts detected exhibit full-length characteristics. Most alternative splicing events discovered by long-read sequencing could be validated by short read data (Supplementary Fig. 1g). By comparing to the isoform annotation reference *(Gencode.v39*), the detected transcripts were categorized into known and novel isoforms with eight subtypes (Fig. 1d). The vicinity of SCL4A2, a gene associated with bile acidification, with 8 novel isoforms and 3 known isoforms identified, in chromosome 7 was selected as an example to illustrate the transcriptomic landscape constructed (Fig. 1e).

Moreover, we performed short-read sequencing of these 17 samples, and then mapped these short reads to our full-length isoforms annotation to quantify them at isoform resolution. To determine if these sequencing data reflect the gallbladder physiological function, we classified genes associated with gallbladder development, bile concentration, acidification, and release.^35–39^ High expression level and isoform diversity could be observed in most of those genes, except for CCKAR and CCKBR which are responsible for gallbladder contraction and bile expulsion to the small intestine carried out by smooth muscle cells not epithelial cells (Supplementary Fig. 1h). These results demonstrated that we generated a high-quality, comprehensive full-length transcript expression dataset with single isoform resolution for gallbladder system. We further investigated the abundant and coding potential of novel transcripts in gallbladder. Approximately 40% of all transcripts, differentially expressed transcripts and transcripts of cancer-associated genes from COSMIC database (version from July, 2023) were novel^40^ (Supplementary Fig. 1i). Furthermore, 19.12% of abundant transcripts, defined as those present in at least one sample at a proportion of 40% or more of the total transcripts for their respective genes, were also novel. To investigate the protein coding potential of novel transcripts, we identified these transcripts based on the open reading frame (ORF) characteristics. In total, 23,435 novel isoforms were categorized into four types: unchanged ORFs with only UTRs changed; NAGNAG isoforms with alterations in the initiation of the first amino acid residue; short ORFs and premature termination codons (PTCs) mediating nonsense-mediated decay (NMD); and protein-coding novel isoforms likely encoding novel long proteins (Fig. 1f). In cancer over-expressed novel isoforms defined by elevating in either cancer tissue or cells compared to normal, 873 (41.14% of all) transcripts were classified into protein-coding novel isoforms (Fig. 1g).

Next, we integrated the annotation and expression of 61,676 full-length transcripts isoforms to construct the GBC transcriptomic atlas that includes 23,435 novel isoforms, 228 fusion transcripts and 5211 differentially expressed isoforms between GBC and normal counterparts (Fig. 1h). We then focused on these protein-coding novel isoforms and found that these encoded proteins were distributed across all subcellular compartments with prominent membrane localization (Fisher’s exact test, *p* < 0.0001) (Fig. 1i, j). This finding was consistent with the observation that transcripts from membrane genes presented with more exons and longer length (Supplementary Fig. 1j, k). Of note, the functional enrichment analysis of these novel protein-coding isoforms revealed that RTK were enriched as one of the top functional categories (Fig. 1k). These results demonstrated that the long-read transcriptomic atlas defined abundant protein-coding transcript isoforms that were overlooked previously but highly functional in GBC, particularly in RTK pathways.

## A novel ERBB2 isoform is overexpressed in cancer cells and associated with cell proliferation and patient survival

**Fig. 2:**
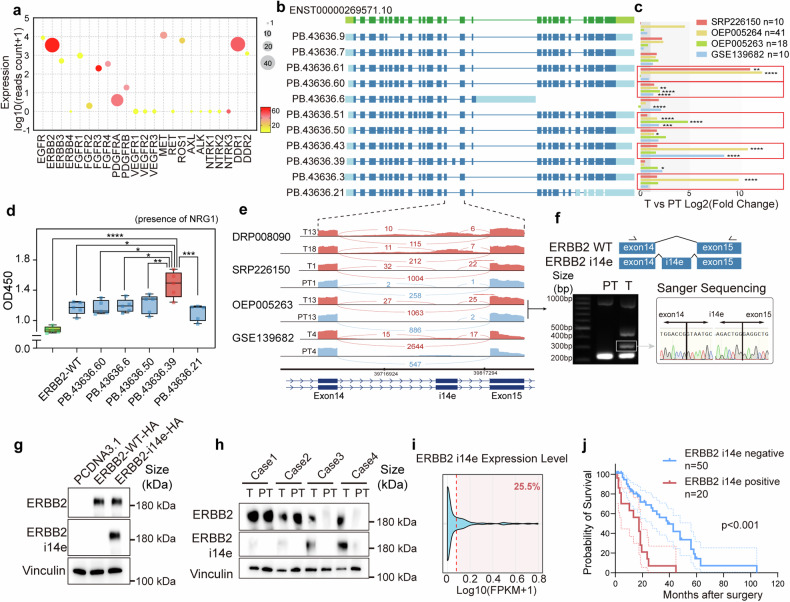
Novel ERBB2 i14e identification and clinical characteristics. **a** Gene expression profile of twenty-three typical receptor tyrosine kinase genes with expression level, total isoform number (size) and novel isoform ratio (color). **b** Structures of eleven selected ERBB2 novel isoform were displayed. **c** Five isoforms were upregulated in four independent GBC RNAseq datasets. Wilcoxon rank sum test was performed. **d** GBC-SD cells transfected with ORF of selected ERBB2 isoforms and subjected CCK8 cell counts (day 7). One-way ANOVA test (*n* = 5) was performed. Data are presented in box-whisker plot. **e** Sashimi plots of ERBB2 at exon14-exon15 segmentation for four typical cases from different GBC RNAseq datasets. **f** Structure of ERBB2 i14e isoform was shown and reverse transcription PCR was performed in one GBC case (T13 and PT13 from OEP005263) with primers (arrows) and the band around 300 bp was extracted and subjected to sanger sequencing. **g** ERBB2-WT and ERBB2 i14e ORF were transfected into GBC-SD cells and western blot was performed using our house-developed anti-ERBB2 i14e antibody to detect ERBB2 i14e, while the commercial anti-ERBB2 antibody recognized both ERBB2-WT and ERBB2 i14e. **h** Four pairs of GBCs with para-tumor tissues were collected for protein detection by western blotting. **i** The distribution of ERBB2 i14e expression was determined using RNAseq data from 137 GBC cases. **j** Prognostic Kaplan–Meier plot of GBC patients’ survival was shown to be associated with ERBB2 i14e protein expression detected by immunohistochemistry. Log-rank (Mantel-Cox) test was performed. T tumor, PT para-tumor

RTKs are transmembrane receptors that play crucial roles in cell biology, including cell proliferation, invasion, and differentiation. Diverse mechanisms of RTK dysfunction have been revealed to initiate carcinogenesis.^6^ Given the hyperactive RTK genes and the enrichment of novel protein-coding isoforms in GBC, we further examined the expression levels and the number of novel and total isoforms of these RTKs genes. Among 23 RTK genes, ERBB2 and DDR1 exhibited the greatest transcriptomic isoform diversity with 37 isoforms identified for each. Notably, 24 of these isoforms for ERBB2 and 19 for DDR1 were novel (Fig. 2a). It has been reported that ERBB2 displays the robust ability to drive GBC development.^11,41^ Therefore, we evaluated the expressions of all 37 ERBB2 transcript variants (Supplementary Fig. 2), including 11 selected novel isoforms (Fig. 2b). Four independent short reads RNA sequencing cohorts involving 137 GBC patients were used to quantify the isoform expression. The result indicated that 5 novel isoforms exhibited significantly higher expression in cancerous tissues compared to corresponding adjacent non-cancerous tissues in paired datasets (Fig. 2c).

To further evaluate the biological functions of these five novel transcripts, the plasmid constructs with CDSs of those isoforms and the wild-type were transfected into GBC-SD cells for proliferation assays. Unexpectedly, the overexpression of these transcripts did not significantly impact cell growth (Supplementary Fig. 3a). The functional defect of these isoforms is likely attributed to the inactive ERBB2 and ERBB3 in the absence of exogenous stimuli, because ERBB2 requires heterodimerization with other members of the ErbB family, such as ERBB3, to initiate downstream signaling. Neuregulin 1 (NRG1) is the ligand for ERBB3 and the activator for ERBB2-ERBB3 heterodimer. In the presence of NRG1, GBC cells carrying ERBB2 isoforms exhibited accelerated cell growth in which one variant (PB.43636.39) gave rise to the strongest induction of cell proliferation (Fig. 2d).

This novel ERBB2 isoform contained a novel 102 bp exon within intron 14 (between exon 14 and 15), termed as ERBB2 intron 14 derived exon (ERBB2 i14e) (Fig. 2b and Supplementary Table 3). The sashimi plots derived from four independent GBC cohorts showed that ERBB2 i14e was elevated in cancer (Fig. 2e). The RT-PCR and sanger sequencing from exon 14 to exon 15 on cDNA samples from OEP005263 validated the expression and sequences of exon i14e transcript (Fig. 2f).

To further identify the protein expression of ERBB2 i14e in GBC, we screened the immunogenicity of the 34 amino acids and selected the potential unique peptide (19 amino acids) from exon i14e to create a specific antibody against only ERBB2 i14e isoform (Supplementary Fig. 3b, c). Western blot analysis using samples overexpressing ERBB2 demonstrated high specificity of this antibody, whereas a commercial ERBB2 antibody recognized the common peptides shared by all isoforms (Fig. 2g and Supplementary Fig. 3d). Our antibody could not bind to other RTK proteins (Supplementary Fig. 3e) and did not affect the proliferation of ERBB2 i14e expressing cells (Supplementary Fig. 3f). We then chose four ERBB2 positive GBC samples and identified at least two cases with high expression of ERBB2 i14e protein (Fig. 2h). Immunofluorescence revealed that ERBB2 i14e was located mainly on the cell membrane (Supplementary Fig. 3g). Subsequently, we found that this novel ERBB2 transcript could be detected in approximately 25.5% of the 137 GBC RNA-seq samples (Fig. 2i). Furthermore, in a cohort of 70 GBC patients, those harboring this novel ERBB2 isoform exhibited significantly shorter overall survival (Fig. 2j). To explore the underlying genetic basis of this novel transcript, we compared the genomic coordinates of the novel exon with reported common mutations in GBC. The results showed that no somatic mutations were detected in the vicinity of the novel exon (Supplementary Fig. 3h), indicating that alternative splicing regulation is more likely accounted for the generation of this isoform. Collectively, our data demonstrated that the novel ERBB2 i14e variant is translatable and its over-expression is associated with elevated cell proliferation and poor patient survival.

## ERBB2 i14e enhances proliferation via ERBB3

**Fig. 3:**
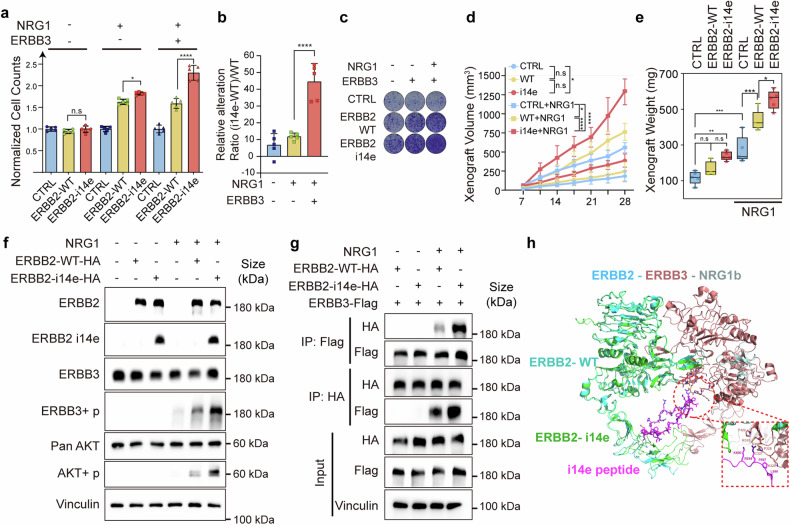
ERBB2 i14e promoted cell proliferation and interact with ERBB3. **a** GBC-SD cells were transfected with ERBB2 WT, ERBB2 i14e and/or ERBB3 in the presence or absence of NRG1 (100 ng/ml) and then subjected to cell counting assays after 7 days. One-way ANOVA test (*n* = 5) was performed. Data are presented as mean ± SD. **b** The difference value between ERBB2 i14e and ERBB2 WT relative to ERBB2 WT was assessed. One-way ANOVA test (*n* = 5) was performed. Data are presented as mean ± SD. **c** Clone formation assays were also performed after culture for 14 days. **d**, **e** Individual xenograft volume and weight were measured after lentivirus infected GBC-SD cells were subcutaneously planted and 1 μg/ml NRG1 was injected into tumors every three days after day 7. One-way ANOVA test (*n* = 5) was performed. xenograft volumes are presented as mean ± SD, and xenograft weight are presented in box-whisker plot. **f** Western blotting on GBC-SD cells transfected with ERBB2 variants and co-cultured with 100 ng/ml NRG1. **g** Coimmunoprecipitation assays were used to examine the interaction between ERBB2 and ERBB3. **h** Presumable interaction between ERBB2 i14e and ERBB3 based on the structure PDB 7MN8. i14e peptides (purple) indicated the peptides generated from intron 14 derived exon

Furthermore, we attempted to decipher molecular mechanisms of ERBB2 i14e in tumor cell growth. We ectopically expressed ERBB2 i14e or wide-type ERBB2 in GBC-SD and ZJU-0430 cell lines and confirmed the comparable overall ERBB2 expression levels in both groups (Supplementary Fig. 4a). Our results showed that ERBB2 i14e induced greater proliferation than ERBB2 WT in the co-expression with ERBB3 and in the presence of NRG1 (Fig. 3a–c and Supplementary Fig. 4b). In line with these in vitro results, subcutaneous cell-derived xenografts assays also indicated that cells overexpressing ERBB2 i14e developed larger volume tumors than the wide type-expressing cells (Fig. 3d, e). To determine the intracellular signaling pathways mediating ERBB2 i14e-induced cell proliferation, we examined AKT activation states as it mediates ERBB2-ERBB3 heterodimer cellular signaling.^42^ As expected, ERBB2 i14e promoted a higher level of phosphorylated ERBB3 and AKT than wild-type ERBB2 (Fig. 3f and Supplementary Fig. 4c) and AKT inhibitors fully abolished the proliferation induced by ERBB2 i14e (Supplementary Fig. 4d). Co-immunoprecipitation experiments further confirmed a more intense interaction between ERBB2 i14e and ERBB3 than the association between wild-type ERBB2 and ERBB3 (Fig. 3g). Additionally, protein structural analyses showed that the i14e peptide is located at the extracellular IV domain of ERBB2 where it served as a putative adaptor to interact with ERBB3 (Fig. 3h). Overall, compared with the wild-type form, ERBB2 i14e possessed stronger capability of interacting with ERBB3, thereby potentially eliciting downstream signaling cascade and promoting the proliferation of GBC cells.

## Acquired expression of ERBB2 i14e leads to trastuzumab resistance

**Fig. 4:**
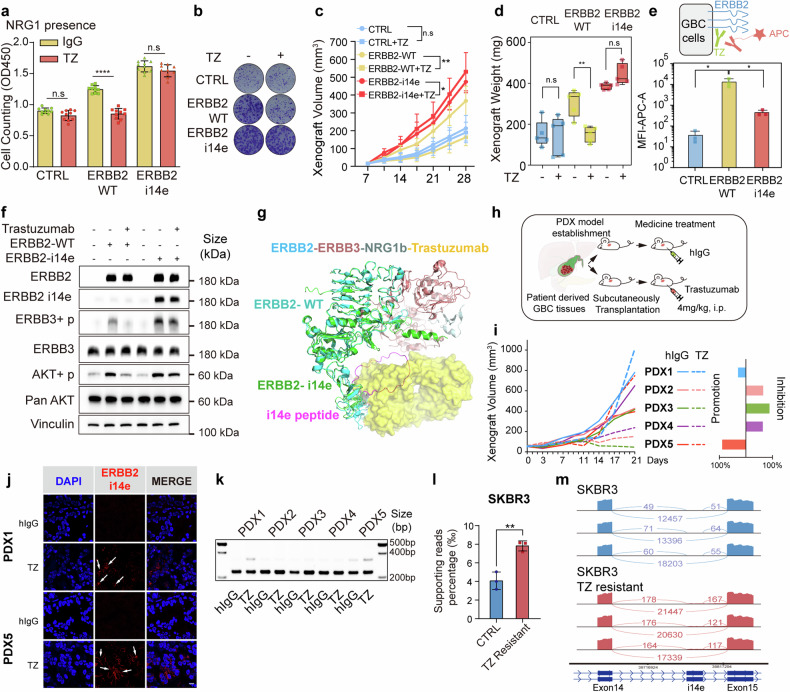
Cells or tumors expressing ERBB2 i14e were resistant to trastuzumab treatment. **a**, **b** Cell proliferation and clone formation of GBC-SD cells expressing ERBB2 wild-type or i14e forms in the presence of 20 μg/ml trastuzumab (TZ) using cell counting assays and clone formation assays. One-way ANOVA test (*n* = 10) was performed. Data are presented as mean ± SD. **c**, **d** Xenografted tumor volume and weight were measured after above GBC-SD cells were subcutaneously planted and 4 mg/kg trastuzumab was intraperitoneally injected twice a week. One-way ANOVA test (*n* = 5) was performed. Xenograft volumes are presented as mean ± SD and xenograft weight are presented in box-whisker plot. **e** Flow cytometry assays on GBC-SD cells expressing ERBB2 variants. Cells were treated with trastuzumab and APC-conjugated anti-human IgG antibody to detect the trastuzumab binding. One-way ANOVA test (*n* = 5) was performed. Data are presented as mean ± SD. **f** AKT phosphorylation was determined by WB in GBC-SD cells expressing ERBB2 WT or ERBB2 i14e in the presence of 20 μg/ml trastuzumab. **g** Presumable interaction between ERBB3 and ERBB2 i14e where peptides of i14e prevented binding of trastuzumab to ERBB2. **h** Patient derived xenografts (PDX) from five gallbladder patients were inoculated into nude mice and trastuzumab was administered intraperitoneally at 4 mg/kg twice a week. **i** Tumor volume was measured and the inhibition or promotion effects on PDX proliferation were shown on the right panel. **j** ERBB2 or ERBB2 i14e expression was determined using immunofluorescence in PDX1 and PDX5 tumors. Scale bar is 10 μm. **k** RT-PCR was also carried out on five PDX samples from Fig. 4i. From GEO dataset (GSE244537), ERBB2 i14e presence was shown by the relative supporting reads ratio in SKRB3 breast cancer control and TZ resistant cells. Two-sided Student’s *t* test (*n* = 5) was performed. **l** and sashimi plots were also exhibited **m**. TZ trastuzumab, i.p. intraperitoneal injection

Trastuzumab is the most therapeutically applied antibody for ERBB2-positive cancers. Mechanically, it suppresses ERBB3 phosphorylation induced by ERBB2, inhibits AKT phosphorylation at both threonine and serine sites, and induces antibody-dependent cell-mediated cytotoxicity (ADCC).^18,43^ Notably, trastuzumab binds to ERBB2 IV domain which is dramatically altered in the ERBB2 i14e form. To interrogate whether the presence of i14e peptide blocks trastuzumab targeting, we first evaluated inhibitory effects of trastuzumab on the proliferation of GBC cells expressing ERBB2 i14e. Colony formation and CCK8 assays exhibited that trastuzumab failed to inhibit the growth of ERBB2 i14e-expressing cells, whereas it effectively suppressed the proliferation of cells expressing wild-type ERBB2 (Fig. 4a, b and Supplementary Fig. 5a, b). In concert with these cultured cell findings, xenografted models in vivo gave rise to identical results where ERBB2 i14e-carrying tumors were irresponsive to trastuzumab therapy (Fig. 4c, d). Flow cytometry analysis showed the decreased binding of trastuzumab to ERBB2 i14e compared with wild-type ERBB2 (Fig. 4e and Supplementary Fig. 5c). Additionally, trastuzumab significantly inhibited ERBB3 and AKT phosphorylation mediated by ERBB2-WT but had no effects on ERBB2 i14e (Fig. 4f and Supplementary Fig. 5d). Protein structure analysis further supported the result of the failure of trastuzumab to access ERBB2 extracellular IV domain where the additional peptide segment of ERBB2 i14e took place the binding interface (Fig. 4g). Collectively, these experimental findings demonstrate that ERBB2 i14e protects cancer cells from trastuzumab binding, leading to resistance to trastuzumab therapy.

To further investigate whether resistance to trastuzumab is ascribed to the acquired expression of ERBB2 i14e, we implanted five GBC PDXs subcutaneously into nude mice and treated them with trastuzumab. It was noted that two out of five (PDX1 and PDX5) showed rapid tumor growth from day14 to 21 during trastuzumab treatment, while the remaining three PDXs exhibited moderate tumor growth (Fig. 4h, i). Accordingly, ERBB2 i14e expression was dramatically increased in trastuzumab-treated tumors compared to IgG-treated tumors as assessment with immunofluorescence and RT-PCR (Fig. 4j, k), suggesting that acquired expression of ERBB2 i14e is accounted for the resistance to trastuzumab.

To investigate whether the expression of ERBB2 i14e contributes to trastuzumab resistance in other types of cancers, not limited to GBC, we assessed ERBB2 i14e levels in a secondary trastuzumab-resistant breast cancer cell line SKBR3^44^ and found identical results with increased ERBB2 i14e (Fig. 4l, m). In a breast cancer dataset comprising 11 samples,^45^ only one patient received trastuzumab treatment and harbored ERBB2 i14e isoform (Supplementary Fig. 5e). A gastric cancer cell line MKN1 cells also exhibited a trend toward increased ERBB2 i14e expression upon trastuzumab treatment^46^ (Supplementary Fig. 5f). In summary, ERBB2 i14e is inducible in adaptation of trastuzumab, thus renders tumors resistant.

## Intervention of ERBB2 i14e increases trastuzumab sensitivity

**Fig. 5:**
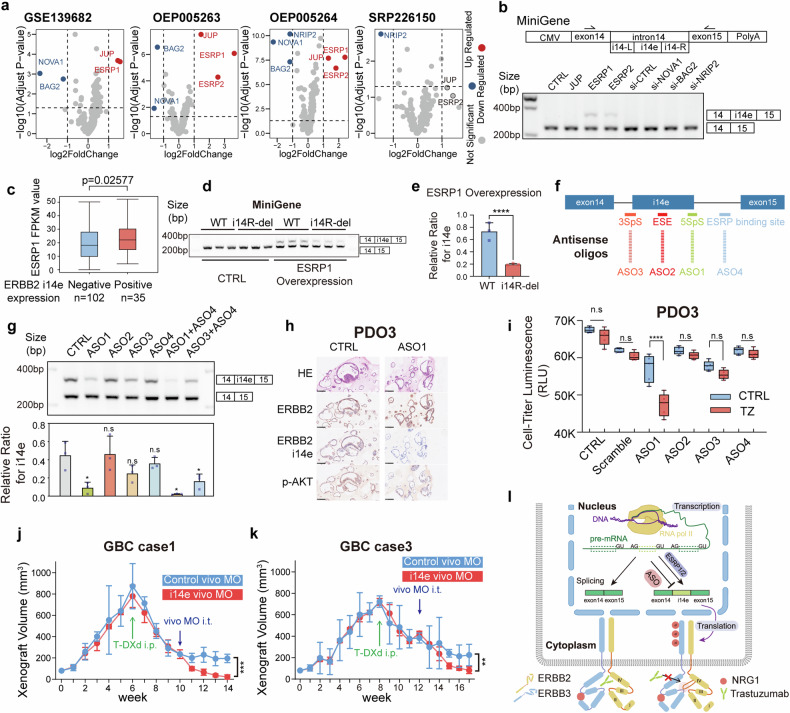
ERBB2 i14e was generated from ESRP1/2 and inhibited by antisense oligonucleotides. **a** Differential expression of RNA splicing factors in four independent GBC RNAseq datasets. **b** The scheme of MiniGene containing ERBB2 exon14 to exon 15 was shown. RT-PCR was performed on GBC-SD cells overexpressing MiniGene when splicing factor expressions were enforced or silenced. **c** The FPKM values of ESRP1 were calculated in groups with or without ERBB2 i14e expression. Wilcoxon rank sum test was performed. Data are presented in box-whisker plot. **d** Transcript splicing generating ERBB2 i14e was determined in GBC-SD cells expressing ESRP1 WT or i14R-deleted mutant by RT-PCR after MiniGene plasmids were transfected. **e** The relative ratio for i14e was calculated and shown. Two-sided student’s *t* test (*n* = 3) was performed. Data are presented as mean ± SD. **f**, **g** ASOs were designed for corresponding RNA splicing sites and applied to GBC PDO3 before RT-PCR detection. One-way ANOVA test (*n* = 3) was performed. Data are presented as mean ± SD. **h** ERBB2, ERBB2 i14e and p-AKT were examined using IHC on paraffin-embedded GBC PDO3. **i** Proliferation of GBC PDO3 cells was determined by cell-titer-glo assays in the presence of ASOs and trastuzumab. One-way ANOVA test (*n* = 5) was performed. Data are presented in box-whisker plot. **j**, **k** Gallbladder cancer PDXs with high ERBB2 expression were implanted to nude mice and when tumor volume reached 800 mm^3^, the administration of T-DXd (10 mg/kg, twice a week) was initiated. Four weeks later, vivo-MO (0.2 mM, twice a week) was added to the treatment. The initiation time for each drug was marked in arrow in figures. Vivo-MO: purple; T-DXd: green. Two-sided Student’s *t* test (*n* = 3) was performed. Data are presented as mean ± SD. **l** Schematic representation of ERBB2 i14e generation, trastuzumab resistance and ASO effects. PDO Patient derived organoid, IHC Immunohistochemistry, ASO Antisense oligonucleotides, i.p. intraperitoneal injection, i.t. intra-tumoral injection, SpS Splicing Site, ESE Exonic Splicing Enhancer

To decipher the molecular mechanisms underlying the induced expression of ERBB2 i14e, we focused on regulatory factors for RNA splicing that may mediate the alternative splicing of ERBB2 i14e exon. We performed differential gene expression analysis for 439 splicing factors in four independent paired GBC RNA datasets,^47,48^ including 79 pairs of GBC tumor and para-tumor tissues. Six factors were identified, including upregulated ESRP1, ESRP2, and JUP, and downregulated NOVA1, BAG2, and NRIP2 (Fig. 5a). Subsequently, we utilized a Minigene plasmid containing exon14-intron14-exon15 to investigate the induction on ERBB2 i14e expression by either overexpression of ESRP1, ESRP2, JUP or knockdown of NOVA1, BAG2, NRIP2 genes, mimicking the differential gene expression in GBC (Supplementary Fig. 6a, b). Overexpression of ESRP1 and ESRP2, but not others, resulted in the induced expression of ERBB2 i14e, suggesting that ESRP1/2 serves as a potential regulator of ERBB2 i14e splicing (Fig. 5b). Moreover, ERBB2 i14e expression was positively correlated with ESRP1 expression in GBC tissues (Fig. 5c). Given that ESRP1/2 bind to UGG-rich regions and promote the inclusion of the upstream exon and exclusion of the downstream exon,^49,50^ we identified a UGG-rich domain located in i14-R at 47-60 bp downstream of i14e exon (Supplementary Fig. 6c). A 14 bp deletion of the UGG-rich region (14R-del) led to decreased expression of ERBB2 i14e when induced by ESRP1 over-expression (Fig. 5d, e), suggesting that this UGG-rich domain in i14R is necessary for the ESRP1 binding and ERBB2 i14e formation.

Of interest, antisense oligonucleotides (ASOs) are widely used to modulate gene expression, particularly for exon skipping.^51,52^ We thus utilized this strategy to determine if ASOs can block the expression of ERBB2 i14e. Four sites were selected for ASO targets: ASO1, 3’ Splicing Site (3SpS); ASO2, exon splicing enhancer sites (ESE); ASO3, 5’ Splicing Site (5SpS) and ASO4, ESRP1/2 binding site (Fig. 5f). One patient-derived organoid (PDO3) with high endogenous expression of ERBB2 i14e and ESRP1 was used to evaluate the inhibition efficacy of ASOs (Supplementary Fig. 7a, b). Among the tested ASOs, ASO1 exhibited the most potent inhibitory effect, while the effect of ASO4 was less pronounced (Fig. 5g). Although ASO4 alone had limited effect, its combination with ASO1 demonstrated a synergistic effect. These results indicate that the 5SpS site is also essential for ERBB2 i14e splicing, while ESRP1 binding site is required but not sufficient for ERBB2 i14e splicing. In the L-2F7 cell line, even with robust ESRP1 overexpression, the efficiency of inducing i14e inclusion remained low, indicating the possible involvement of additional factors in the regulation of i14e (Supplementary Fig. 7c). Furthermore, we observed a similar regulatory role of ESRP1 in i14e generation in both SKBR3 and MKN1 cell lines which has been shown to generate ERBB2 i14e, suggesting a broader conservation of this regulatory mechanism across different cancer types (Supplementary Fig. 7c). In PDO3 cells, ASO1 treatment decreased the phosphorylation level of AKT and enhanced trastuzumab-mediated growth suppression (Fig. 5h, i).

Utilizing PDX models of GBC, we investigated the efficacy of a sequential treatment regimen combining trastuzumab deruxtecan (T-DXd), a highly promising ADC based on trastuzumab, and vivo-morpholino oligos (vivo-MO), a type of ASO active in vivo. The addition of i14e vivo-MO targeting the 5SpS alone did not have inhibitory effects on tumor (Supplementary Fig. 7d); but notably, the combination of early treated T-DXd with i14e vivo-MO gave rise to more therapeutic effects on tumors than that with control vivo-MO in ERBB2 positive GBC cases (Fig. 5j, k and Supplementary Fig. 7e). The results highlight the T-DXd-resistance mediated by ERBB2 i14e in tumor development. RNA-seq analysis of PDXs following treatment demonstrated ERBB2 i14e suppression was correlated with reduced tumor cell proliferation, enhanced apoptosis, and altered cellular redox state (Supplementary Fig. 7f). Altogether, our results demonstrate that the novel exon of ERBB2 i14e, generated through alternative splicing involving ESRP1, contributes to trastuzumab resistance (Fig. 5l). Antisense oligonucleotides targeting the 5’ splicing sites of ERBB2 i14e can sensitize trastuzumab therapy, suggesting that combined treatment of ERBB2 i14e ASOs with trastuzumab or trastuzumab-drug conjugates holds great promising benefit for cancer patients expressing ERBB2 i14e.

## Discussion

As a connecting component in central dogma, RNA research could offer a multi-dimensional perspective to investigate intracellular abnormalities, particularly in protein-coding genes in cancer. And alternative splicing in cancer due to various reasons have been proved to profoundly influence the tumor progression and carcinogenesis. However, the prevalent short reads sequencing fails to recapitulate the full landscape of RNA owe to its insufficient read technology. To date, the long-read sequencing has been developed as a powerful tool to successfully identify a large amount of novel transcript isoforms with unprecedented accuracy. This innovation has stimulated great interests to uncover novel genes and transcripts that contribute to cancer growth and evolution.^53,54^ Given its extraordinary efficacy, the long reads transcriptome sequencing can unexpectedly discover a number of unexplored genes that potentially drive malignant transformation of GBC. As a highly aggressive but poorly understood malignancy, GBC patients often lose their chance for surgical treatment at late stages and unfortunately, they also don’t have well-established targeted therapeutic guidance for the lack of studies.^1,55^ Therefore, the application of long-read transcriptome sequencing to GBC research can establish a promising avenue to elucidate the underlying molecular mechanisms and identify novel therapeutic targets for GBC patients.

Hence, we took advantage of long-read sequencing of cancer and donor normal samples together with gallbladder cell lines to build up the full-length transcriptome atlas of GBC. This transcriptome atlas reflects the basic function of gallbladder and highlights the transcriptome diversity of highly activated RTKs (Fig. 1k). By screening tumor-specific, highly-expressed transcripts in RTK genes, we identified a novel isoform with a novel exon derived from intron14, named ERBB2 i14e (Fig. 2c). And the analysis of patient cohorts revealed that this variant was detectable in approximately 25.5% of GBC cases (Fig. 2i) and associated with worse prognosis (Fig. 2j). This variant considerably facilitated the interaction between ERBB2 and ERBB3 and triggered stronger downstream signaling cascade than the wild type ERBB2 to promote cancer cell proliferation. Further studies indicated that ERBB2 i14e could also endow cancer cells with irresponsiveness to trastuzumab, one of the most important targeted drugs for ERBB2 used in clinical treatment for breast cancer for over 20 years. In GBC, ERBB2 represents the most promising therapeutic target for GBC at present and the clinical trials with trastuzumab^14,56–58^ for the most promising means have begun but remain to be evaluated in future. Given that ERBB2 i14e is inducible in our PDX xenografts, it should be taken particularly account for the acquired trastuzumab resistance during the treatment. The present findings may provide mechanistic explanation for the trastuzumab resistance if a population of ERBB2-positive patients do not respond well to trastuzumab. Not limited to GBC, identical induction of ERBB2 i14e was also be recapitulated in breast cancer, thus indicating that the expression and function of ERBB2 i14e could be ubiquitous across varied epithelial cancer types. Protein structure analysis enabled us to fundamentally understand the action mechanism of the ERBB2 i14e. The ERBB2 i14e harbors extra 34 amino acids on the extracellular IV domain, which facilitates the heterodimerization with ERBB3 and forms steric hindrance to prevent trastuzumab binding.

We further discovered that ESRP1/2 binds to a UGG-rich motif proximal 3’end of the i14e exon, promoting its inclusion in the mature mRNA (Fig. 5d). It is of interest that our experiments revealed a less pronounced effect when inhibiting solely the ESRP1 binding site (ASO4) compared to the 5’ splice site (ASO1) (Fig. 5g). It is still plausible, given the complex nature of RNA splicing involving multiple factors, that other factors binding to the 5’ splice site are essential to regulate the RNA splicing event. In addition, the combination experiments using ASO1 and ASO4, or ASO3 and ASO4, revealed a synergistic effect on ERBB2 i14e suppression, indicating that the ESRP1 binding site is required but not sufficient for ERBB2 i14e splicing. Another intriguing finding was the limited ability of ESRP1 to induce i14e splicing in non-cancerous gallbladder epithelial cell line (L-2F7), which suggests other tumor-intrinsic factors are also involved in ERBB2 i14e splicing (Supplementary Fig. 7c). Combined treatment with ASOs and trastuzumab in organoids, as well as with vivo-MO and T-DXd in PDX models, significantly inhibited tumor cell growth, highlighting that combined therapy can improve the anti-ERRB2 targeting efficacy, particularly in overcoming ERBB2 resistance (Fig. 5j, k). It is noteworthy that the in vitro models endogenously expressing high levels of ERBB2 i14e are crucial for this research, particularly for those experiments involving ASOs inhibition. But most GBC cell lines doesn’t meet the standard (Supplementary Fig. 7a). Fortunately, we have made significant progress in developing GBC organoids and screened one invaluable model for further research. Nevertheless, the development of more in vitro models in the future will provide more chances to explore the downstream mechanisms in GBC.

Due to the novel variant ERBB2 i14e, we developed the first antibody specific for this novel protein. As for the specificity of this rabbit antibody, we initially identified a specific peptide (SLPRIKLGGGPRGRGHRDW) sequence as a unique immune antigen predicted for an unvalidated ERBB2 isoform based on NCBI BLAST analysis. Then, we transfected six ERBB2 variants and some RTK genes individually to cells to overexpress protein and found that only ERBB2 i14e-expressing cells were specifically detected for a positive signal with this antibody (Supplementary Fig. 3d, e). However, this antibody could not affect on cell proliferation directly (Supplementary Fig. 3f), indicating that it could specifically bind to ERBB2 i14e, but does not have neutralizing function. Our future research will focus on developing monoclonal neutralizing antibodies and ADCs targeting ERBB2 i14e and investigate the role of this isoform in other ERBB2 associated cancer types, ultimately enhancing the efficacy of trastuzumab-based regimens for ERBB2 i14e-expressing cancers.

In summary, our study has presented a typical example to demonstrate the translational and clinically therapeutic value of this full-length transcriptome atlas in the development of cancer and drug resistance (Fig. 5l). Discovering ERBB2 i14e as a novel isoform of ERBB2 has mechanistically shed lights on cancer cell proliferation and drug resistance, pointing to a diagnostic biomarker and therapeutic target for GBC.

## Methods

### Sample collection, library preparation and sequencing

Normal gallbladder epithelium tissues were donated by organ donors after consents and evaluated as pathologically normal. Informed consents of GBC patient tissues with corresponding clinical data were reviewed and approved by Shanghai Jiao Tong University School of Medicine, Renji Hospital Ethics Committee. Tissue samples were stored and grinded in liquid nitrogen. Total RNA was extracted using the RNeasy Mini Kit (QIAGEN), quantified with the Qubit RNA BR Assay Kit (Molecular Probes), and quality-checked using a 2100 Agilent Bioanalyzer (RIN ≥ 7.5). For long read sequencing, first-strand cDNA was synthesized using the SMARTer PCR cDNA Synthesis Kit (Clontech, 634925) according to the manufacturer’s protocol. Subsequently, 12 cycles of PCR amplification were conducted with PrimeSTAR GXL DNA polymerase (Clontech, R050A). The amplified cDNA products were used to generate SMRTbell template libraries following the Iso-Seq protocol by Pacific Biosciences (SMRTbell Template Prep Kit 1.0). Finally, sequencing was performed on the PacBio Sequel II System.

For short read sequencing, total RNA was purified using an oligo(dT) magnetic bead-based protocol to enrich for poly(A) mRNA. The enriched mRNA was then fragmented into smaller pieces using fragmentation buffer. First-strand cDNA synthesis was primed with random hexamers, followed by second-strand cDNA synthesis. The double-stranded cDNA was purified using AMPure XP beads and subjected to end repair, A-tailing, and adapter ligation. Size selection of the ligation products was performed using AMPure XP beads, and the final cDNA library was enriched by PCR amplification. The quality and quantity of the constructed libraries were assessed using Qubit 2.0 fluorometer and Agilent 2100 Bioanalyzer. qPCR was employed to accurately determine the effective concentration of the libraries. Qualified libraries were subsequently subjected to paired-end (PE150) sequencing on a high-throughput sequencing platform (Hiseq2000, Illumina).

### Hi-Fi reads acquisition with long read sequencing

Iso-Seq (v3.8.2) pipeline was applied. Briefly, CCS reads were generated with CCS (v6.2.0). Primers and SMRT adapters were removed using Lima (v2.2.0) for full-length (FL) reads generation, followed by removal of artificial concatemers reads and trimming of poly(A) tails in Iso-Seq3 Refine. SMRTlink dataset was used to integrate all involved samples and generate combined data, with Iso-Seq3 Cluster that determined high-quality transcripts with default parameters.

The high-quality (HQ) data was mapped to human (GRCh38) reference genome using Pbmm2 (v1.8.0). Isoseq3 collapse was used to remove redundant isoforms. Filtering of transcripts with low reads count supports (lower than 3) and 5’ terminal degraded isoforms were accomplished by Cupcake with default parameters. Sqanti3 (v5.5.1) was used to characterize and further filter the transcripts that were likely artifacts.

The transcriptome atlas isoforms were visualized by wiggleplot, ggtranscripts and txviz (https://github.com/wwei-lab/txviz/), an R package for visualizing exon and intron structures of different transcripts.

### 5’CAGE peaks and polyA annotation analysis

During the transcript assembly process, two key files were used provided by SQANTI3: one containing multiple polyA tail motifs (e.g., AATAAA and ATTAAA) and another specifying the TSS (transcription start site) regions of the human genome to evaluate the 5’ and 3’ of transcripts. For the 5’ of transcripts identified, we calculated whether they located in 50 bp of any annotated 5’ TSS from GAGE data. For 3’ of transcripts identified, we calculated whether there are annotated polyA tail motifs in 50 bps. Finally, the results for four categories of transcripts (full-splice_match: aligns perfectly with the reference genome; incomplete-splice_match, shows partial alignment with the reference genome; novel_in_category, a novel occurrence within a known splicing category; novel_not_in_category, represents a previously unidentified splicing pattern) were demonstrated. After assembly, TSS and TES (transcription termination site) information from the reference genome (e.g., Gencode v39) were extracted and aligned the assembled transcripts to these reference sites. The closest distance was selected for statistical analysis including density plotting.

### Depth saturation analysis

Sequencing data was randomly subsampled 10%, 30%, 50%, 70%, 90% and 100% of the total reads using samtools. The resulting BAM files were subsequently processed through the Cupcake and SQANTI3 pipelines to identify both known and novel transcripts. Through curve fitting of the relationship between the number of detected transcripts and sequencing depth, we estimated saturation point.

### Gene or Isoform quantification

The quality control of illumina RNA-seq data was accomplished by FastQC (v0.12.1). Then RNAseq data trimmed by fastp (v0.23.4) with default parameters. Then the clean data was aligned to the human (GRCh38) reference genome using STAR (v2.7.10b) with two-pass-Mode. Samtools (v1.17) was used to sort and index bam files.

For transcript expression quantification, gtf file of all samples was generated from Sqanti3 and StringTie (v1.3.1c) was used along with individual bam file to calculate expression for each possible transcript. This read-count information was extracted directly from the files created by StringTie using a Python script (prepDE.py). Differential gene expression analysis was performed with the R package DESeq2.

For data obtained from external sources, GSE244537, GSE75688 and GSE141352 were downloaded from GEO database. Raw fastq data were filtered using fastp to obtain clean data. Subsequently, reads were aligned to the hg38 reference genome using STAR in two-pass mode. Quantification was performed using stringtie. For splicing factor analysis, the splicing factor genes were listed in Supplementary Table 4. The number of reads supporting the ERBB2 i14e splice variant was counted from the STAR-generated SJ.out.tab file. To visualize and analyze reads in more detail, the Integrative Genomics Viewer (IGV) was used.

### ORF annotation with NMD classification and GO enrichment analysis

TranSuite (V 0.2.2) was used to identify the multiple NMD-related signals and the coding potential of long-read transcripts. According to four criteria including Coding_potentiality, Features, Alternative_ORF, and NMD_features, we reclassified transcripts into two major classes and five subcategories. Under the coding classification, they were divided into three subcategories: Unchanged ORF, NAGNAG, and protein-coding novel isoform. The unproductive classification was more challenging, as a transcript often exhibited multiple features. To simplify the classification, we prioritized short ORFs, while the remaining types, including uORF, ldORF, long_3UTR, etc., were classified as PTC types since they tend to simultaneously meet the conditions for premature termination codons. Gene ontology (GO) analysis was performed with Metascape (http://metascape.org) in this study. Visualization was implemented with R.

### Protein structure analysis

In this study, a subset of protein structure was analyzed utilizing the ColabFold software, guided by AlphaFold2. Specifically, the structural information for ERBB2-ERBB3 heterodimer was obtained from previously published research,^59^ with the PDB accession code 7MN8 as a reference. The HDOCK software was employed to simulate protein-protein interactions, and PyMOL software was utilized to generate visual representations for observational purposes.

### Cell and organoid culture

Cell lines including NOZ, GBC-SD, ZJU-0430, OCUG1, SKBR3, and MKN1 were authenticated by short tandem repeats assays (STR) and tested mycoplasma free. Those cells were cultured in DMEM medium with 10% FBS and L-2F7 cells were cultured according to previous study.^33^ Cancer cells derived from GBC patients were cultured in the form of organoids, following the methods described in previously published literature.^60^

### Antibody generation for ERBB2 i14e

A peptide specific for the ERBB2 i14e was selected based on its immunogenicity using Protean Software. To facilitate conjugation to keyhole limpet hemocyanin (KLH), a cysteine residue was added to the N-terminus of the peptide. The synthesized peptide (CSLPRIKLGGGPRGRGHRDW) was quality controlled by mass spectrometry (MS) and high-performance liquid chromatography (HPLC).

New Zealand white rabbits were immunized four times, once per week, with 0.5 mg of KLH-conjugated peptide (CSLPRIKLGGGPRGRGHRDW). Serum was collected after the third and fourth immunizations for titer determination. Titer was measured using indirect ELISA. Subsequently, the antibody was purified using the antigen as an affinity matrix, followed by dialysis and concentration. Finally, the purified antibody was subjected to ELISA for titer and concentration determination, and SDS-PAGE analysis for purity assessment.

### Cell growth assays

For cell counting assays, 1000 cells were seeded in a well of 96-well-plates. Relative cell counts were evaluated by CCK8 kit (40203ES88, Yeasen, Shanghai, China). Five hundred cells were cultured in six-well-plates for ten to fourteen days before being stained by crystal violet and pictured as clone formation assays. For organoids, 5000 cells were counted, suspended in 5 μl Matrigel (356231, Corning, USA) and seeded into 96-well-plates. Two weeks later, the viability was detected by Cell Titer-Glo kit (C0065, Beyotime, Shanghai, China).

### Immunohistochemistry and Immunofluorescence

Paraffin-embedded tissues were sectioned on a microtome (3 μm thickness) and collected on glass slides. For immunohistochemistry (IHC), after being baked at 65 °C for 30 min, the slides were placed on BOND-RXm (Leica, Australia) and incubated with antibodies. Stained tissues were photographed under a microscope (BX43, Olympus, Tokyo, Japan).

For immunofluorescence (IF) experiments, standard procedures were followed. Deparaffinization, antigen retrieval using alkaline solutions, and blocking with 5% Goat Serum (C0265, Beyotime, Shanghai, China) were performed. After incubating with the primary antibody at 4 °C for 8 h, incubation with the secondary antibody was carried out for 2 h at 37 °C. Finally, slides were sealed with mounting medium after staining nuclei with DAPI. Following staining completion, observation and image acquisition were conducted using a fluorescence confocal microscope (FV3000, Olympus, Tokyo, Japan).

### Plasmid, siRNA and ASO

The sequences of siRNAs and ASOs were listed in Supplementary Table 2. The backbone of ASO is 2-OMe-modified and for in vivo assays, vivo-morpholino oligos synthesized by Gene Tools (OR, USA) were used. To create ectopic expression constructs, CDS regions of ERBB2 WT, ERBB2 i14e, ERBB3, ESRP1, ESRP2, JUP, ERBB3 and others were amplified by PCR from cDNA and subsequently cloned into the pCDNA3.1 vector. The cloned constructs were transformed using serial seamless cloning kits (Vazyme, Nanjing, China).

### Minigene associated assays

Genomic DNA was extracted from NOZ cells and used for PCR to amplify the sequence from exon14 to exon15, and clone to PCDNA3.1 vector digested by *NheI* and *BamHI* (Thermofisher FastDigest) with CloneEXpress II One Step Cloning Kit (Vazyme, C112). After the recombinant clone was generated, the ligation mixture was transformed into DH5α cells and plated on LB agar containing ampicillin. Individual colonies were picked, cultured before plasmid DNA was extracted for Sanger sequencing validation. For the splicing factor perturbation assays, 1 μg plasmids of Minigene were transfected to in vitro cultured cells along with other 1 μg overexpression plasmid; ASO or siRNA were transfected 24 h later. After 48–72 h from the first transfection, total RNA was extracted using Trizol, followed by DNase digestion and reverse-transcription into cDNA. After 37 cycles of PCR using specific primer (Supplementary Table 2), the products were electrophoresed on a 2.5% agarose gel for 25 min and visualized using a DNA imager. Finally, the band intensities were quantified using ImageJ and the relative ratio for ERBB2 i14e was calculated by dividing the grayscale value of the i14e containing band by that of the other band.

### Cell transfection and lentivirus infection

Lipofectamine 3000 (L3000075, ThermoFisher, MA, USA) was used for siRNA, ASO and plasmid transfection according to the manufacturer’s instructions. For organoid transfection, after Matrigel was digested by TrypLE, 400 μl transfection mix solution was prepared at final concentration of 100 nM for ASO and the ratio of ASO to Lipo3000 was 1 pmol:1.5 μl. The solution was then used to resuspend 5 × 10^4^ organoid cells, followed by centrifugation at 600 *g* for 1 h and subsequent incubation at 37 °C for 3 h. Then transfected organoid cells were collected, suspended in fresh Matrigel and cultured according to standard organoid culture protocols.

Second generation lentivirus package system were used to generate lentivirus consisting of pLVX, pMD2.G and psPAX2. After infecting cells for 48 h, puromycin was applied to select positive populations for three days (GBC-SD: 20 μg/ml; ZJU-0430: 15 μg/ml).

### Western blot and Co-immunoprecipitation

For Western blot experiments, a standardized procedure and method were employed. High molecular weight proteins such as ERBB2 were analyzed using 7.5% polyacrylamide gel. For Co-IP experiments, we use plasmids to express tag-fused proteins in cell lines. After cell lysis with protein lysis buffer and centrifugation at 15000 *g*, the supernatant was then mixed with magnetic beads conjugated with anti-FLAG or anti-HA antibodies (Bimake, TX, USA). After incubation at 4 °C for 8 h, the beads were washed twice with PBS. Then beads were resuspended with 1× SDS loading buffer and denatured at 95 °C for 10 min. After the magnetic beads were removed, Co-IP samples were prepared for subsequent Western blotting. The primary and secondary antibodies were listed in the Supplementary Table 1.

### Flow cytometry

After being transfected with ERBB2 variant plasmid, 5 × 10^5^ GBC cells were collected. Cells were then treated with 20 μg/ml Trastuzumab (T9912, TargetMol, MA, USA) at 4 °C for an hour. After washing with PBS for three times, APC-conjugated anti-human IgG (34813ES60, Yeasen, Shanghai, China) was added and incubated for 30 min at 4 °C. Then samples were incubated with 7-AAD (559925, BD Biosciences, NJ, USA) at 4 °C for 20 min and analyzed by FACS Celesta Flow Cytometer (BD Bioscience). Finally, FACS data were analyzed using FlowJo software (version 10; BD Life Sciences).

### In vivo animal assays

For cell-derived xenograft (CDX) assays, 1 × 10^6^ GBC cells of GBC-SD or ZJU-0430 cell lines were collected and injected subcutaneously into nude mice (nu/nu, 6- to 8-week-old females). For PDX assays, patient derived GBC tissues were collected, digested into single-cell suspension, and transplanted into nude mice subcutaneously. Then, the PDX tissues were further passaged, and drug administration commenced at the F2 generation. Trastuzumab and T-DXd was injected intraperitoneally twice a week at dose of 4 mg/kg and 10 mg/kg twice a week respectively. For multipoint intratumoral injection, 1 μg/ml NRG1 was used every three days and 0.2 mM vivo-morpholino oligos were injected twice a week.

### Figure construction and statistics analysis

GraphPad Prism v9 and R were used for data analysis and to generate figures. Statistical analysis included but not limited to Student’s *t* test, one-way ANOVA, Mann–Whitney U test, Chi-square were used. Figures were composed and standardized by Adobe illustrator 2019. P-values less than 0.05 were considered statistically significant. Sample sizes and statistical analysis methods were indicated in figures or figure legends.

## Supplementary information

Supplementary Materials

## Data Availability

All data has been uploaded in NGDC database (https://ngdc.cncb.ac.cn/?lang=en). The transcriptome atlas was based on long and short read sequencing data from eight gallbladder cancer (GBC) samples and four normal gallbladder samples (HRA007861), as well as five gallbladder cell lines (HRA008115). The database accession numbers of two independent GBC NGS RNAseq cohort are HRA007859 and HRA007860. Intermediate files including stablished gtf file, raw read data and quantification table were deposited at OMIX007982. All the data could be accessible upon corresponding authors’ approval for reasonable requests without prejudice to approved ethics and local legislation via NGDC. The critical bioinformatic code has been uploaded to github link: https://github.com/wwei-lab/GBC-Long-read-Transcriptome/tree/main.

## References

[1] Roa, J. C. et al. Gallbladder cancer. *Nat. Rev. Dis. Prim.* **8**, 69 (2022). 36302789 10.1038/s41572-022-00398-y PMC12314663

[2] Valle, J. W., Kelley, R. K., Nervi, B., Oh, D. Y. & Zhu, A. X. Biliary tract cancer. *Lancet* **397**, 428–444 (2021). 33516341 10.1016/S0140-6736(21)00153-7

[3] Song, X. et al. Overview of current targeted therapy in gallbladder cancer. *Signal Transduct. Target Ther.* **5**, 230 (2020). 33028805 10.1038/s41392-020-00324-2 PMC7542154

[4] Casaletto, J. B. & McClatchey, A. I. Spatial regulation of receptor tyrosine kinases in development and cancer. *Nat. Rev. Cancer* **12**, 387–400 (2012). 22622641 10.1038/nrc3277 PMC3767127

[5] Du, Z. & Lovly, C. M. Mechanisms of receptor tyrosine kinase activation in cancer. *Mol. Cancer* **17**, 58 (2018). 29455648 10.1186/s12943-018-0782-4 PMC5817791

[6] Saraon, P. et al. Receptor tyrosine kinases and cancer: oncogenic mechanisms and therapeutic approaches. *Oncogene* **40**, 4079–4093 (2021). 34079087 10.1038/s41388-021-01841-2

[7] Tomuleasa, C. et al. Therapeutic advances of targeting receptor tyrosine kinases in cancer. *Signal Transduct. Target Ther.* **9**, 201 (2024). 39138146 10.1038/s41392-024-01899-w PMC11323831

[8] Hynes, N. E. & Lane, H. A. ERBB receptors and cancer: the complexity of targeted inhibitors. *Nat. Rev. Cancer* **5**, 341–354 (2005). 15864276 10.1038/nrc1609

[9] Hynes, N. E. & MacDonald, G. ErbB receptors and signaling pathways in cancer. *Curr. Opin. Cell Biol.* **21**, 177–184 (2009). 19208461 10.1016/j.ceb.2008.12.010

[10] Li, M. et al. Whole-exome and targeted gene sequencing of gallbladder carcinoma identifies recurrent mutations in the ErbB pathway. *Nat. Genet.* **46**, 872–876 (2014). 24997986 10.1038/ng.3030

[11] Li, M. et al. Genomic ERBB2/ERBB3 mutations promote PD-L1-mediated immune escape in gallbladder cancer: a whole-exome sequencing analysis. *Gut* **68**, 1024–1033 (2019). 29954840 10.1136/gutjnl-2018-316039

[12] Yang, P. et al. Somatic genetic aberrations in gallbladder cancer: comparison between Chinese and US patients. *Hepatobiliary Surg. Nutr.* **8**, 604–614 (2019). 31929987 10.21037/hbsn.2019.04.11 PMC6943012

[13] Kiguchi, K. et al. Constitutive expression of ErbB-2 in gallbladder epithelium results in development of adenocarcinoma. *Cancer Res.* **61**, 6971–6976 (2001). 11585718

[14] Javle, M. et al. HER2/neu-directed therapy for biliary tract cancer. *J. Hematol. Oncol.* **8**, 58 (2015). 26022204 10.1186/s13045-015-0155-z PMC4469402

[15] Hainsworth, J. D. et al. Targeted therapy for advanced solid tumors on the basis of molecular profiles: results from MyPathway, an Open-Label, Phase IIa multiple basket study. *J. Clin. Oncol.* **36**, 536–542 (2018). 29320312 10.1200/JCO.2017.75.3780

[16] Hyman, D. M. et al. HER kinase inhibition in patients with HER2- and HER3-mutant cancers. *Nature* **554**, 189–194 (2018). 29420467 10.1038/nature25475 PMC5808581

[17] Meric-Bernstam, Funda et al. Single agent activity of ZW25, a HER2-targeted bispecific antibody, in heavily pretreated HER2-expressing cancers. *J. Clin. Oncol.* **36**, 2500 (2018).

[18] Valabrega, G., Montemurro, F. & Aglietta, M. Trastuzumab: mechanism of action, resistance and future perspectives in HER2-overexpressing breast cancer. *Ann. Oncol.* **18**, 977–984 (2007). 17229773 10.1093/annonc/mdl475

[19] Maadi, H., Soheilifar, M. H., Choi, W.-S., Moshtaghian, A. & Wang, Z. Trastuzumab Mechanism of Action; 20 Years of Research to Unravel a Dilemma. *Cancers* **13**, 3540 (2021). 34298754 10.3390/cancers13143540 PMC8303665

[20] Bartsch, R., Wenzel, C. & Steger, G. G. Trastuzumab in the management of early and advanced stage breast cancer. *Biologics* **1**, 19–31 (2007). 19707345 PMC2721347

[21] Friedlaender, A. et al. EGFR and HER2 exon 20 insertions in solid tumours: from biology to treatment. *Nat. Rev. Clin. Oncol.* **19**, 51–69 (2022). 34561632 10.1038/s41571-021-00558-1

[22] Anido, J. et al. Biosynthesis of tumorigenic HER2 C-terminal fragments by alternative initiation of translation. *EMBO J.* **25**, 3234–3244 (2006). 16794579 10.1038/sj.emboj.7601191 PMC1500971

[23] Maadi, H., Nami, B., Tong, J., Li, G. & Wang, Z. The effects of trastuzumab on HER2-mediated cell signaling in CHO cells expressing human HER2. *BMC Cancer* **18**, 238 (2018). 29490608 10.1186/s12885-018-4143-x PMC5831215

[24] Lewis, G. D. et al. The HER2-directed antibody-drug conjugate DHES0815A in advanced and/or metastatic breast cancer: preclinical characterization and phase 1 trial results. *Nat. Commun.* **15**, 466 (2024). 38212321 10.1038/s41467-023-44533-z PMC10784567

[25] Ferraro, E., Drago, J. Z. & Modi, S. Implementing antibody-drug conjugates (ADCs) in HER2-positive breast cancer: state of the art and future directions. *Breast Cancer Res.* **23**, 84 (2021). 34380530 10.1186/s13058-021-01459-y PMC8356386

[26] Cortés, J. et al. Trastuzumab deruxtecan versus trastuzumab emtansine in HER2-positive metastatic breast cancer: long-term survival analysis of the DESTINY-Breast03 trial. *Nat. Med.* **30**, 2208–2215 (2024). 38825627 10.1038/s41591-024-03021-7 PMC11333275

[27] Zhang, M. et al. An inflammatory checkpoint generated by IL1RN splicing offers therapeutic opportunity for KRAS-mutant intrahepatic cholangiocarcinoma. *Cancer Discov.* **13**, 2248–2269 (2023). 37486241 10.1158/2159-8290.CD-23-0282

[28] Sun, Q. et al. Long-read sequencing reveals the landscape of aberrant alternative splicing and novel therapeutic target in colorectal cancer. *Genome Med.* **15**, 76 (2023). 37735421 10.1186/s13073-023-01226-y PMC10512518

[29] Wang, Y. et al. rMATS-turbo: an efficient and flexible computational tool for alternative splicing analysis of large-scale RNA-seq data. *Nat. Protoc.* **19**, 1083–1104 (2024). 38396040 10.1038/s41596-023-00944-2

[30] Ameur, A., Kloosterman, W. P. & Hestand, M. S. Single-molecule sequencing: towards clinical applications. *Trends Biotechnol.* **37**, 72–85 (2019). 30115375 10.1016/j.tibtech.2018.07.013

[31] van Dijk, E. L., Jaszczyszyn, Y., Naquin, D. & Thermes, C. The third revolution in sequencing technology. *Trends Genet.* **34**, 666–681 (2018). 29941292 10.1016/j.tig.2018.05.008

[32] Ermini, L. & Driguez, P. The application of long-read sequencing to cancer. *Cancers* **16**, 1275 (2024). 38610953 10.3390/cancers16071275 PMC11011098

[33] Wang, Z. et al. Establishment and characterization of an immortalized epithelial cell line from human gallbladder. *Front. Oncol.* **12**, 994087 (2022). 36387215 10.3389/fonc.2022.994087 PMC9650220

[34] Huang, K. K. et al. Long-read transcriptome sequencing reveals abundant promoter diversity in distinct molecular subtypes of gastric cancer. *Genome Biol.* **22**, 44 (2021). 33482911 10.1186/s13059-021-02261-x PMC7821541

[35] Kruse, E., Uehlein, N. & Kaldenhoff, R. The aquaporins. *Genome Biol.* **7**, 206 (2006). 16522221 10.1186/gb-2006-7-2-206 PMC1431727

[36] Trampert, D. C., van de Graaf, S. F. J., Jongejan, A., Oude Elferink, R. P. J. & Beuers, U. Hepatobiliary acid-base homeostasis: Insights from analogous secretory epithelia. *J. Hepatol.* **74**, 428–441 (2021). 33342564 10.1016/j.jhep.2020.10.010

[37] Wank, S. A. Cholecystokinin receptors. *Am. J. Physiol.* **269**, G628–G646 (1995). 7491953 10.1152/ajpgi.1995.269.5.G628

[38] Hunter, M. P. et al. The homeobox gene Hhex is essential for proper hepatoblast differentiation and bile duct morphogenesis. *Dev. Biol.* **308**, 355–367 (2007). 17580084 10.1016/j.ydbio.2007.05.028 PMC2045067

[39] Delous, M. et al. Sox9b is a key regulator of pancreaticobiliary ductal system development. *PLoS Genet.* **8**, e1002754 (2012). 22719264 10.1371/journal.pgen.1002754 PMC3375260

[40] Sondka, Z. et al. The COSMIC Cancer Gene Census: describing genetic dysfunction across all human cancers. *Nat. Rev. Cancer* **18**, 696–705 (2018). 30293088 10.1038/s41568-018-0060-1 PMC6450507

[41] Zhang, Y. et al. Single-cell RNA-sequencing atlas reveals an MDK-dependent immunosuppressive environment in ErbB pathway-mutated gallbladder cancer. *J. Hepatol.* **75**, 1128–1141 (2021). 34171432 10.1016/j.jhep.2021.06.023

[42] Holbro, T. et al. The ErbB2/ErbB3 heterodimer functions as an oncogenic unit: ErbB2 requires ErbB3 to drive breast tumor cell proliferation. *Proc. Natl Acad. Sci. USA* **100**, 8933–8938 (2003). 12853564 10.1073/pnas.1537685100 PMC166416

[43] Vernieri, C. et al. Resistance mechanisms to anti-HER2 therapies in HER2-positive breast cancer: Current knowledge, new research directions and therapeutic perspectives. *Crit. Rev. Oncol. Hematol.* **139**, 53–66 (2019). 31112882 10.1016/j.critrevonc.2019.05.001

[44] Duan, N. et al. Unveiling alterations of epigenetic modifications and chromatin architecture leading to lipid metabolic reprogramming during the evolutionary Trastuzumab Adaptation of HER2-Positive Breast Cancer. *Adv. Sci.* **11**, 2309424 (2024). 10.1002/advs.202309424 PMC11095153 38460162

[45] Wang, J., Xu, R., Yuan, H., Zhang, Y. & Cheng, S. Single-cell RNA sequencing reveals novel gene expression signatures of trastuzumab treatment in HER2+ breast cancer: a pilot study. *Medicine* **98**, e15872 (2019). 31261495 10.1097/MD.0000000000015872 PMC6617483

[46] Ebert, K. et al. Determining the effects of trastuzumab, cetuximab and afatinib by phosphoprotein, gene expression and phenotypic analysis in gastric cancer cell lines. *BMC Cancer* **20**, 1039 (2020). 33115415 10.1186/s12885-020-07540-7 PMC7594334

[47] Du, J.-X. et al. Splicing factors: Insights into their regulatory network in alternative splicing in cancer. *Cancer Lett.* **501**, 83–104 (2021). 33309781 10.1016/j.canlet.2020.11.043

[48] Beusch, I. et al. Targeted high-throughput mutagenesis of the human spliceosome reveals its in vivo operating principles. *Mol. Cell* **83**, 2578–2594.e2579 (2023). 37402368 10.1016/j.molcel.2023.06.003 PMC10484158

[49] Derham, J. M. & Kalsotra, A. The discovery, function, and regulation of epithelial splicing regulatory proteins (ESRP) 1 and 2. *Biochem. Soc. Trans.* **51**, 1097–1109 (2023). 37314029 10.1042/BST20221124 PMC11298080

[50] Peart, N. J. et al. The global Protein-RNA interaction map of ESRP1 defines a post-transcriptional program that is essential for epithelial cell function. *iScience* **25**, 105205 (2022). 36238894 10.1016/j.isci.2022.105205 PMC9550651

[51] Matsuo, M. Antisense oligonucleotide-mediated exon-skipping therapies: precision medicine spreading from duchenne muscular dystrophy. *JMA J.* **4**, 232–240 (2021). 34414317 10.31662/jmaj.2021-0019 PMC8355726

[52] Veltrop, M. & Aartsma-Rus, A. Antisense-mediated exon skipping: taking advantage of a trick from Mother Nature to treat rare genetic diseases. *Exp. Cell Res.* **325**, 50–55 (2014). 24486759 10.1016/j.yexcr.2014.01.026

[53] Jarvis, E. D. et al. Semi-automated assembly of high-quality diploid human reference genomes. *Nature* **611**, 519–531 (2022). 36261518 10.1038/s41586-022-05325-5 PMC9668749

[54] Liao, W. W. et al. A draft human pangenome reference. *Nature* **617**, 312–324 (2023). 37165242 10.1038/s41586-023-05896-x PMC10172123

[55] Schmidt, M. A., Marcano-Bonilla, L. & Roberts, L. R. Gallbladder cancer: epidemiology and genetic risk associations. *Chin. Clin. Oncol.* **8**, 31 (2019). 31484487 10.21037/cco.2019.08.13

[56] Lavingia, V., Thummar, V. & Mehta, P. Addition of trastuzumab emtansine (T-DM1) in a human epidermal growth factor receptor 2-overexpressed metastatic carcinoma of the gallbladder patient to enhance survival: a case study. *SAGE Open Med. Case Rep.* **10**, 2050313X221137447 (2022). 36467008 10.1177/2050313X221137447 PMC9709179

[57] May, M. et al. Prolonged response to HER2-Directed therapy in three patients with HER2-Amplified metastatic carcinoma of the biliary system: case study and review of the literature. *Oncologist* **26**, 640–646 (2021). 33896096 10.1002/onco.13800 PMC8342570

[58] Wang, J. N. & Xu, B. H. Targeted therapeutic options and future perspectives for HER2-positive breast cancer. *Signal Transduct. Target Ther.* **4**, 34 (2019). 31637013 10.1038/s41392-019-0069-2 PMC6799843

[59] Diwanji, D. et al. Structures of the HER2-HER3-NRG1beta complex reveal a dynamic dimer interface. *Nature* **600**, 339–343 (2021). 34759323 10.1038/s41586-021-04084-z PMC9298180

[60] Yuan, B. et al. Patient-derived organoids for personalized gallbladder cancer modelling and drug screening. *Clin. Transl. Med.* **12**, e678 (2022). 35075805 10.1002/ctm2.678 PMC8786696

