# Supplementary material for: Full-length transcriptome atlas of gallbladder cancer reveals trastuzumab resistance conferred by ERBB2 alternative splicing
Source: Signal Transduct Target Ther. 2025 Feb 14;10:54. doi: 10.1038/s41392-025-02150-w (PMC11825701; doi:10.1038/s41392-025-02150-w)

**Supplementary Materials for**

Full-length transcriptome atlas of gallbladder cancer reveals trastuzumab resistance conferred by ERBB2 alternative splicing

Ziyi Wang, Li Gao, Ziheng JIA, Liguo Liu, Ao Gu, Zhaonan Liu, Qin Zhu, Yichen Zuo, Mingjie Yang, Shijia Wang, Jiyao Ma, Jingyun Zhang, Shimei Qiu, Zhizhen Li, Jinghan Wang, Dongxi Xiang, Fatao Liu, Rong Shao, Yanjing Li, Maolan Li, Wu Wei, Yingbin Liu

Corresponding address: LIU Yingbin: [laoniulyb@shsmu.edu.cn](mailto:laoniulyb@shsmu.edu.cn);

WEI Wu: [wuwei@lglab.ac.cn](mailto:wuwei@lglab.ac.cn); LI Maolan: [limaolan6@163.com](mailto:limaolan6@163.com);

LI Yanjing: [liyanjing@sjtu.edu.cn](mailto:liyanjing@sjtu.edu.cn);

**This File includes:**

Supplementary Figure 1-7

Supplementary Tables 1-4

Uncropped Images of Western Blots

**Supplementary Figure 1**


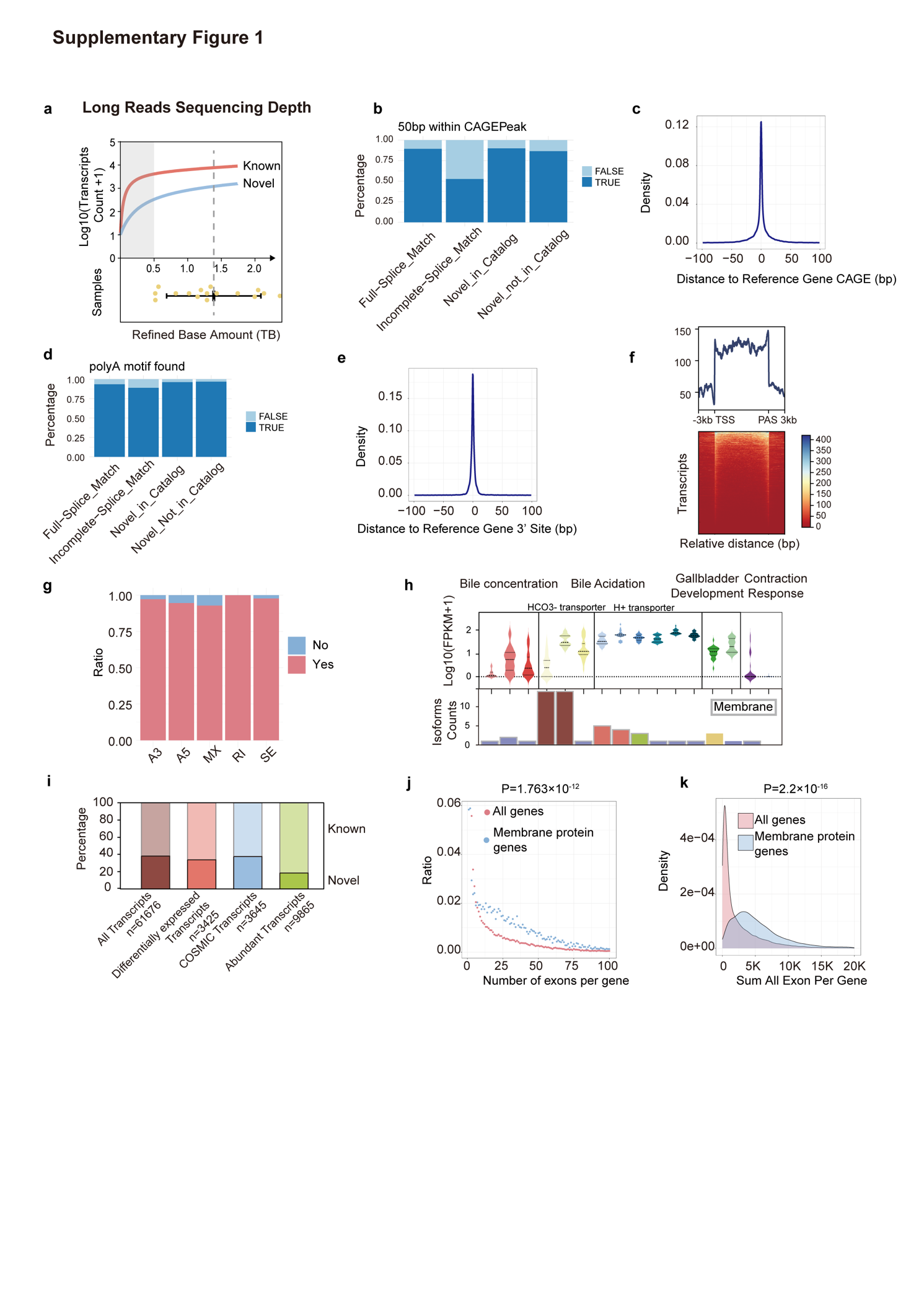


**Quality control of Sequencing Data**

(a) The correlations between sequencing depths and number of detected transcripts (top). Sequencing data amounts of each sample were also shown, and dots exceeding the coordinate range were marked on the edge of the graph (bottom). The quality control metric for isoforms within 50bp CAGE peaks (b&c) or with polyA motif (d&e). (f) The distribution of the distances between assembled transcripts and the nearest annotated TSS (transcription start site) and TES (transcription termination site) based on the Gencode v39 reference genome. GBC-SD_rep1 was used for illustration. (g) Split-reads from second-generation sequencing were used to validate alternative splicing events generated by long read sequencing data. (h) Expression patterns of genes related to inherent gallbladder function: absolute expression levels (top) and the number of isoforms of individual gene (bottom). Genes encoding membrane-localized proteins were outlined in gray. (i) Proportion of known and novel transcripts in different transcript types. (j&k) the comparison of exon number and transcript length between membrane protein coding genes and all genes (Wilcoxon rank sum test).

**Supplementary Figure 2**


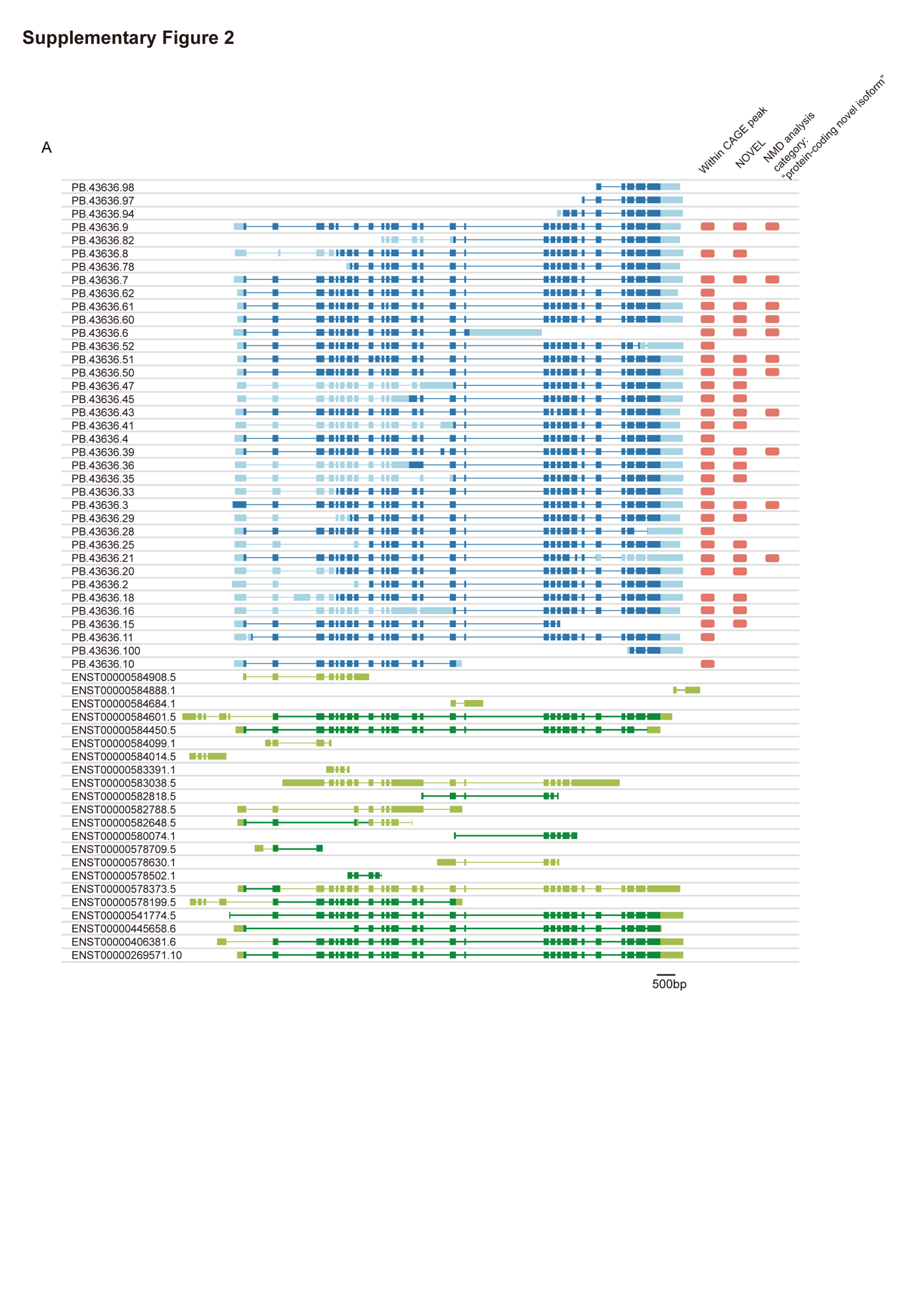


**Transcript profiling of ERBB2 in gallbladder transcriptome atlas**

All filtered ERBB2 isoforms from Hi-Fi reads were shown in blue, in which dark blue indicates the predicted ORF. Twenty-two isoforms in green were annotated in *Gencode* database, in which dark green stood for ORF region. Layered filtering of isoforms were exhibited in read marks from isoform quality control, novelty and ORF classification from Nonsense Mediated Decay (NMD) analysis. The length of intron was not shown in actual size, the scale bar for exons is 500bp.

**Supplementary Figure 3**


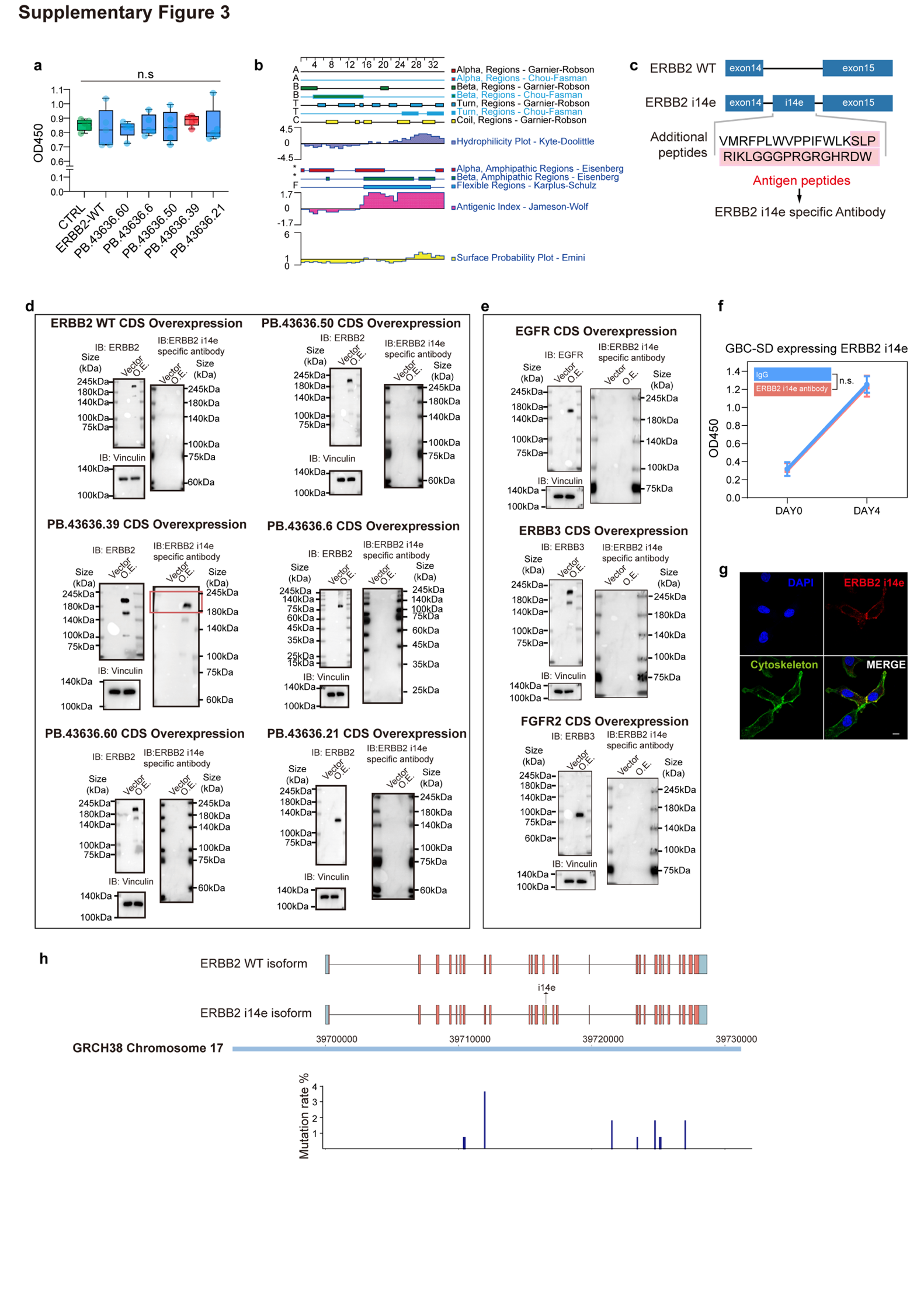


**Construction and validation of the antibody targeting ERBB2 i14e**

(a) After GBC-SD cells were transfected to express various ERBB2 variant ORFs, cell proliferation was measured by CCK8 assays at day 7. One-way ANOVA test (n=5) was performed. Data are presented as box-whisker plot. (b) The immunogenicity analysis of the 34 extra amino acids peptide of ERBB2 i14e was performed using Protean Software. (c) The simplified graph illustrated antigen selection at i14e region for the development of ERBB2 i14e-specific antibody. (d) Six ERBB2 variants were transfected to NOZ cells and subjected to immunoblotting with a commercial ERBB2 antibody and an ERBB2 specific antibody. (e) RTK genes (including EGFR, ERBB3 and FGFR2) were transfected into cells to assess the specificity of ERBB2 i14e antibody. (f) Cell counts assays were performed on GBC-SD cells overexpressing ERBB2 i14e in the presence of IgG or ERBB2 i14e antibody (10μg/ml) for four days. Two-sided student’s *t* test (n=5) was performed. (g) Immunofluorescence of GBC-SD cells overexpressing ERBB2- i14e. was performed. Blue: DAPI; Green: ERBB2 i14e; Red: cytoskeleton (phalloidine). Scale bar is 10 μm. (h) The ERBB2 mutation sites were marked onto ERBB2 i14e transcript structure to evaluate the potential underlying genomic basis.

**Supplementary Figure 4**

**
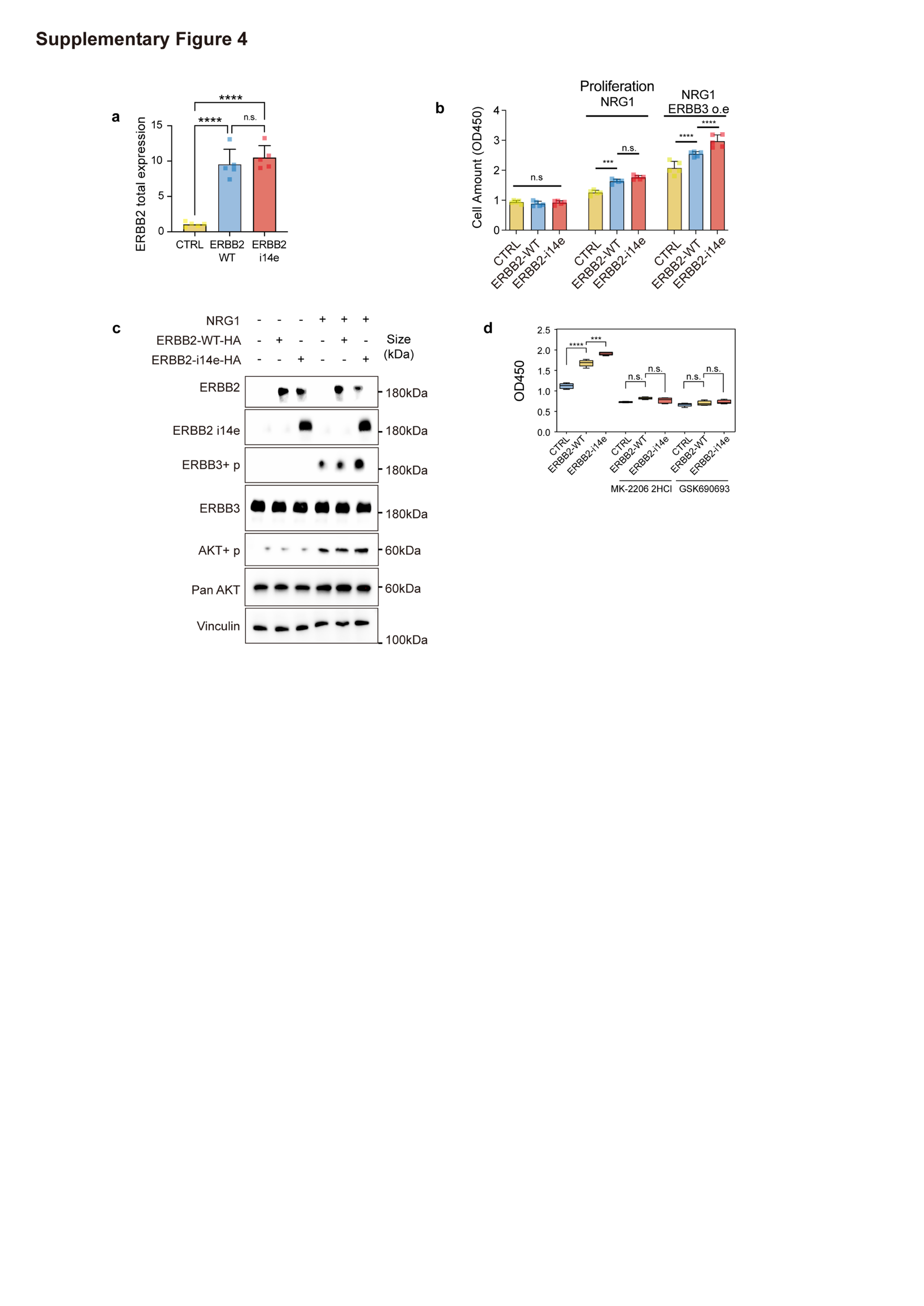
**

**ERBB2 i14e promotes cell proliferation through the AKT pathway**

(a) q-RT PCR was performed on GBC-SD cells transfected with ERBB2 to evaluate the overall ERBB2 expression. One-way ANOVA test (n=10) was performed. Data are presented as mean±SD. (b) ZJU-0430 cell proliferation was measured using cell counting after cells were transfected to express ERBB2 wild-type or i14e forms in the presence of 100 ng/ml NRG1. One-way ANOVA test (n=5) was performed. Data are presented as mean±SD. (c) The phosphorylation of ERBB3 and AKT in ZJU-0430 cells were determined in the presence of NRG (100 ng/ml) for 2 hours using western blotting. (d) ERBB2-expressing GBC-SD cells were subjected to CCK8 cell proliferation assays after treatment with AKT inhibitors (10 μM MK-2206 2HCL and 10 μM GSK690693) for 5 days. One-way ANOVA test (n=5) was performed. Data are presented as mean±SD.

**Supplementary Figure 5**

**
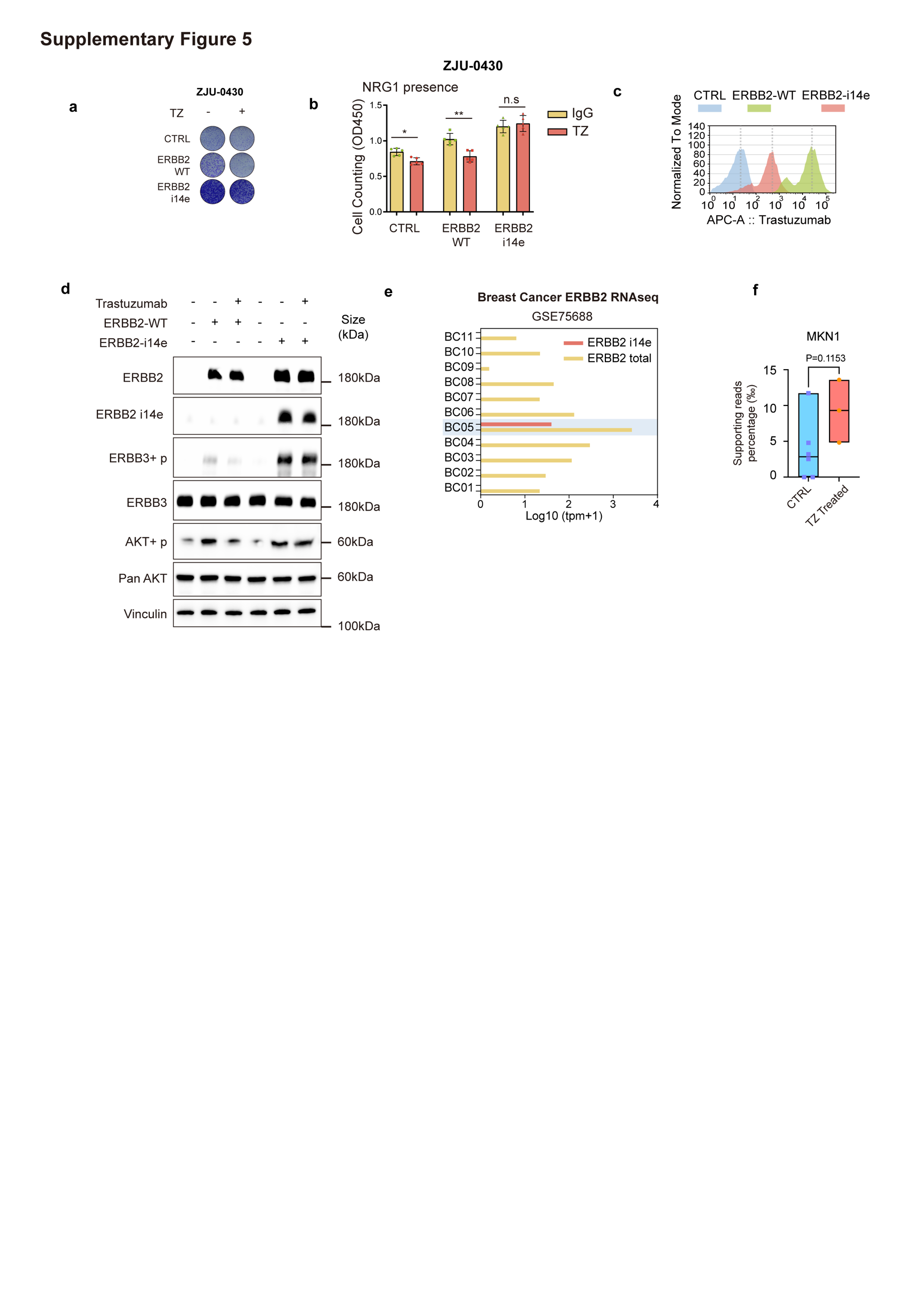
**

**ERBB2 i14e is resistance to trastuzumab**

(a&b) ZJU-0430 cell growth was evaluated by clone formation and CCK8 assays after treatment with 20 μg/ml trastuzumab for 10 days. One-way ANOVA test (n=5) was performed. Data are presented as mean±SD. (c) Binding of trastuzumab to ERBB2 WT or i14e were measured using flow cytometry assays. (d) ERBB3 and AKT phosphorylation in ZJU-0430 cells were determined in the presence of trastuzumab using western blotting. The quantification of ERBB2 total amount and ERBB2 i14e level in 11 breast cancer cases from GSE75688 datasets (e). The relative supporting reads percentage for ERBB2 i14e was calculated in gastric cancer cell line MKN1 from GSE141352 (f). Two-sided student’s *t* test was performed (n=6 vs n=3). Data are presented in floating bars (min to max).

**Supplementary Figure 6**


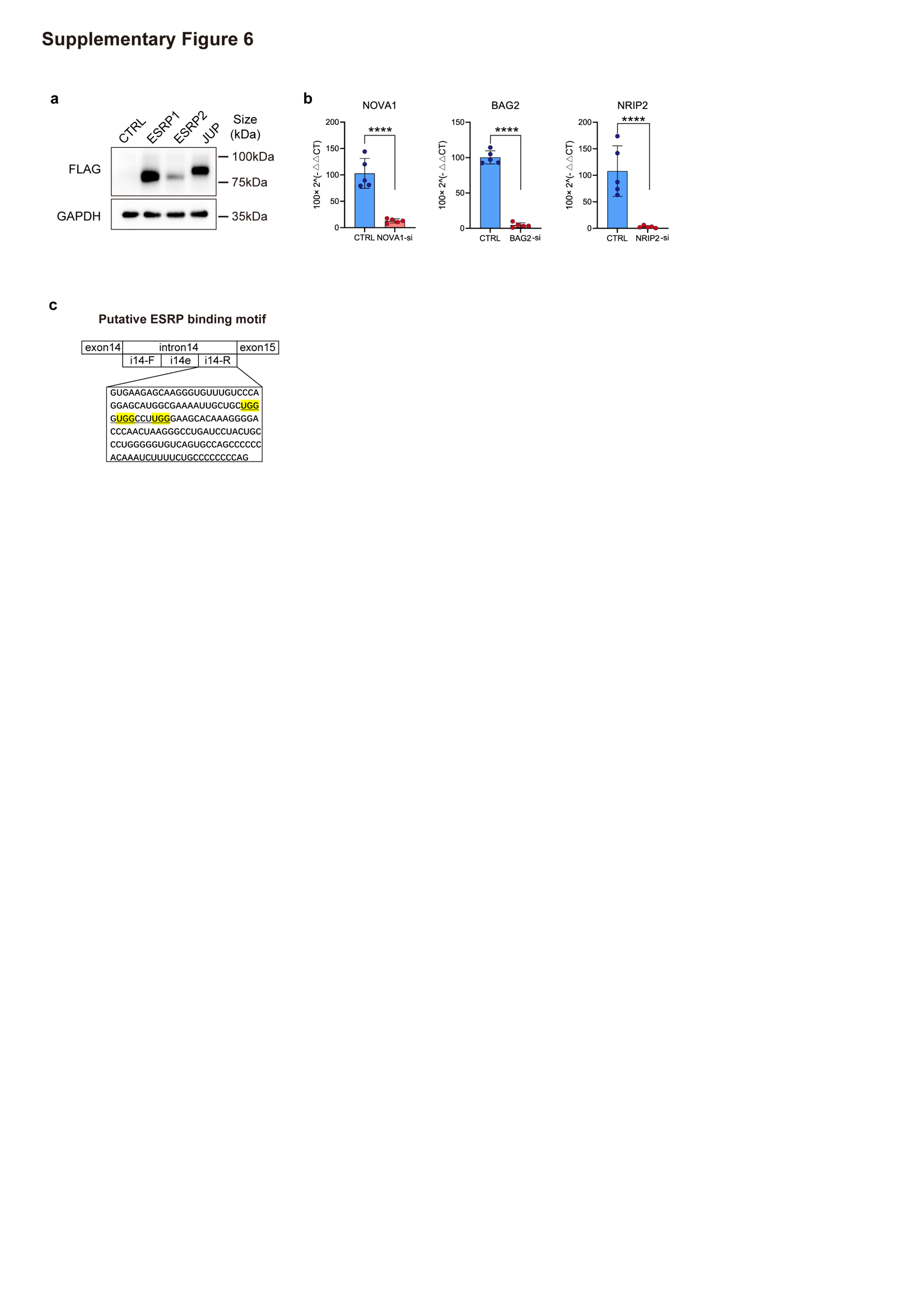


**ESRP1 is identified as a facilitator of i14e inclusion**

(a) Overexpression of ESRP1, ESRP2 and JUP was measured by immunoblotting. (b) Gene knockdown of NOVA1, BAG2 and NRIP2 was quantified by qPCR. Two-sided Student's *t* test (n=5) was performed. Data are presented as mean±SD. (c) The putative binding motif of ESRP1/2 located at ERBB2 i14R was shown.

**Supplementary Figure 7**

**
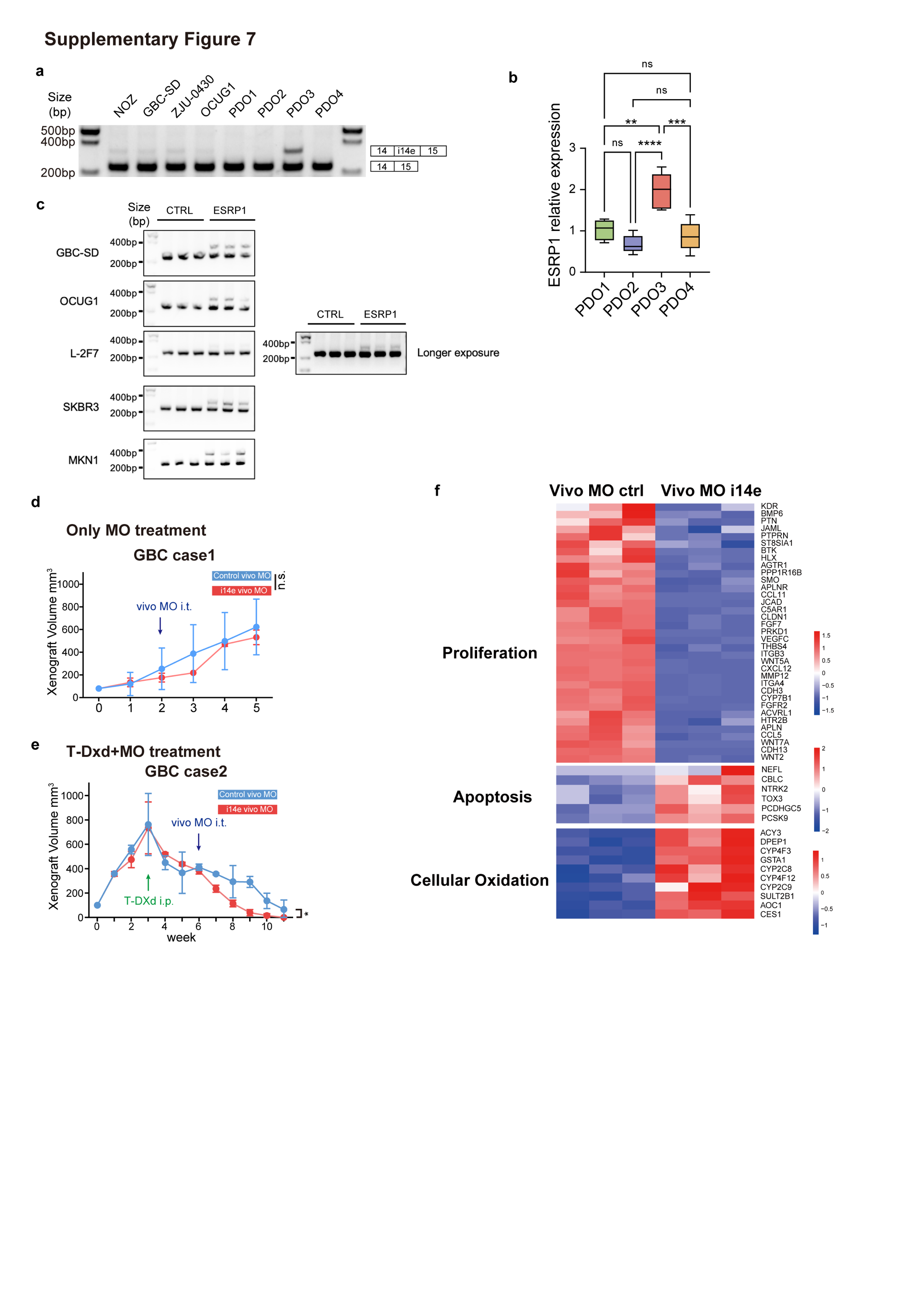
**

**Drug resistance mediated by ERBB2 i14e is attenuated by Antisense Oligonucleotides**

(a) Endogenous levels of ERBB2 i14e were determined by RT-PCR in four gallbladder cancer cell lines and three gallbladder patient-derived organoids. (b) Quantitative PCR was performed to examine the ESRP1 expression in four GBC organoids. Data are presented as a box-whisker plot. (c) Minigene were transfected into various cell lines along with ESRP1 overexpression plasmid, followed by RT-PCR to examine the i14e splicing events.(d) Vivo MO was applied in GBC PDX and the volume were measured every week. Two-sided student’s t test (n=3) was performed. Data are presented as mean±SD. (e) T-DXd was applied on GBC PDX from case 2 and three weeks later vivo-MO was used. The tumor volume changes were measured and plotted. Two-sided student’s t test (n=3) was performed. Data are presented as mean±SD. (f) RNA-seq was performed on PDX samples post-T-DXd treatment followed by in vivo MO therapy. Gene expression related to proliferation, apoptosis, DNA damage and cell oxidation was shown.

**Supplementary Table1**

| **Antibody** | | |
| --- | --- | --- |
| Anti-ERBB2 | Cell signaling technology | 2165 |
| Anti-ERBB2 | Proteintech | 60311-1-Ig |
| Anti-phospho-ERBB2 | Cell signaling technology | 2243 |
| Anti-ERBB3 | Proteintech | 10369-1-Ap |
| Anti-phospho-ERBB3 | Cell signaling technology | 4791 |
| Anti-Vinculin | Proteintech | 66305-1-Ig |
| Anti-HA | Cell signaling technology | 3724 |
| Anti-Flag | Proteintech | 66008-4-Ig |
| Anti-AKT | Cell signaling technology | 4691 |
| Anti-AKT phosphor | Cell signaling technology | 9271 |
| Anti-GAPDH | Proteintech | 60004-1-Ig |
| Trastuzumab | TargetMol | T9912 |
| Trastuzumab Deruxtecan (T-DXd) | Bidepharm | BD01353073 |
| Human IgG | Selleckchem | A2052 |
| **Recombinant Protein and Chemicals** | | |
| MK-2206 2HCL | Selleckchem | S1078 |
| GSK690693 | Selleckchem | S1113 |
| Neuregulin 1 | Abcam | ab282401 |

**Supplementary Table2**

**Primer for PCR**

|  | **Forward** | **Reverse** |
| --- | --- | --- |
| ERBB2  (exon14-exon15) | ATGTGAATGCCAGGCACTGT | GGTGCAGTTGATGGGGCAA |
| **For qPCR** |  |  |
| ERBB2 | GCAGGATATCCAGGAGGTGC | TCAAGATCTCTGTGAGGCTTCG |
| NOVA1 | CCAAGAGGACCAATACGGGC | AGCTGTCAGCACCTGGGTA |
| BAG2 | AGCTGGAGCTCAGGGTTGA | CTTGCTGCTGGGGGTTTCTAA |
| NRIP2 | CGTTGACACAGGCACCCAATA | GGTCGATGCAGCACTTGAGAG |
| **Sequence of siRNA** | | |
|  | **Sense** | **Anti-sense** |
| NOVA1 | UACGAAUUCUCCUUUUUUGTT | CAAAAAAGGAGAAUUCGUATT |
| BAG2 | UCUAUAUCCACUUAUAGUCTT | GACUAUAAGUGGAUAUAGATT |
| NRIP2 | ACAAUACAAGCUUAAGCUCTT | GAGCUUAAGCUUGUAUUGUTT |
| **Sequence of ASO** | | |
| **In vitro ASO** | |  |
| ASO-CTRL | GCGAGCAUCCUUGAUAGCCUAGC |  |
| ASO1 | CUUGCUCUUCACCCAGUCUCUGUG |  |
| ASO2 | AGCUUGAUCCUGGGCAGGGACUU |  |
| ASO3 | AAAACGCAUUACCUAAGGUGUAAC |  |
| ASO4 | GCUUCCCAAGGCCACCCAGCAGCA |  |
| **Vivo Morpholino Oligos** | |  |
| Ctrl | CCTCTTACCTCAGTTACAATTTATA |  |
| I14E | AAACACCCTTGCTCTTCACCCAGTC |  |

**Supplementary Table3**

| Genomic coordinates version: GRCH38 | | | |
| --- | --- | --- | --- |
| **chromosome** | **start** | **end** | **exon number** |
| chr17 | 39700064 | 39700311 | 1 |
| chr17 | 39706990 | 39707141 | 2 |
| chr17 | 39708321 | 39708534 | 3 |
| chr17 | 39709318 | 39709452 | 4 |
| chr17 | 39709813 | 39709881 | 5 |
| chr17 | 39710086 | 39710201 | 6 |
| chr17 | 39710340 | 39710481 | 7 |
| chr17 | 39711928 | 39712047 | 8 |
| chr17 | 39712322 | 39712448 | 9 |
| chr17 | 39715286 | 39715359 | 10 |
| chr17 | 39715446 | 39715536 | 11 |
| chr17 | 39715740 | 39715939 | 12 |
| chr17 | 39716301 | 39716433 | 13 |
| chr17 | 39716515 | 39716605 | 14 |
| **chr17** | **39717067** | **39717168** | **i14e** |
| chr17 | 39717320 | 39717480 | 15 |
| chr17 | 39719787 | 39719834 | 16 |
| chr17 | 39723319 | 39723457 | 17 |
| chr17 | 39723538 | 39723660 | 18 |
| chr17 | 39723912 | 39724010 | 19 |
| chr17 | 39724726 | 39724911 | 20 |
| chr17 | 39725049 | 39725204 | 21 |
| chr17 | 39725327 | 39725402 | 22 |
| chr17 | 39725707 | 39725853 | 23 |
| chr17 | 39726562 | 39726659 | 24 |
| chr17 | 39726815 | 39727003 | 25 |
| chr17 | 39727295 | 39727547 | 26 |
| chr17 | 39727689 | 39728658 | 27 |

**Supplementary Table4**

| ACIN1 | LSM3 | | SF3A3 | | YJU2 | SFRS13A |
| --- | --- | --- | --- | --- | --- | --- |
| ALYREF | LSM4 | | SF3B1 | | ZMAT2 | SFRS2 |
| AQR | LSM5 | | SF3B2 | | ZNF830 | SFRS3 |
| BCAS2 | LSM6 | | SF3B3 | | A2BP1 | SFRS4 |
| BUB3 | LSM7 | | SF3B4 | | CUGBP1 | SFRS5 |
| BUD13 | LSM8 | | SF3B5 | | CUGBP2 | SFRS6 |
| BUD31 | LUC7L3 | | SF3B6 | | DAZAP1 | SFRS7 |
| C9orf78 | MAGOH | | SLU7 | | ELAVL1 | SFRS9 |
| CACTIN | MAGOHB | | SMNDC1 | | ELAVL2 | SYNCRIP |
| CCAR1 | MFAP1 | | SMU1 | | ELAVL3 | TARDBP |
| CCDC12 | MTREX | | SNIP1 | | ELAVL4 | TIA1 |
| CD2BP2 | NKAP | | SNRNP200 | | ESRP1 | TIAL1 |
| CDC40 | NOSIP | | SNRNP27 | | ESRP2 | TRA2A |
| CDC5L | PHF5A | | SNRNP40 | | FMR1 | TRA2B |
| CDK11A | PLRG1 | | SNRNP70 | | FUS | YBX1 |
| CHERP | PPIE | | SNRPA | | Gene | ZRANB2 |
| CRNKL1 | PPIG | | SNRPA1 | | HNRNPA0 | PRPF40A |
| CTNNBL1 | PPIH | | SNRPB | | HNRNPA1 | RBM23 |
| CWC15 | PPIL1 | | SNRPB2 | | HNRNPA2B1 | RBM39 |
| CWC22 | PPIL2 | | SNRPC | | HNRNPA3 | ILF3 |
| CWC25 | PPIL3 | | SNRPD1 | | HNRNPC | ILF2 |
| CWC27 | PPWD1 | | SNRPD2 | | HNRNPD | ZC3H18 |
| DDX23 | PQBP1 | | SNRPD3 | | HNRNPF | SRRT |
| DDX39B | PRCC | | SNRPE | | HNRNPH1 | SRSF1 |
| DDX41 | PRKRIP1 | | SNRPF | | HNRNPH2 | SRSF7 |
| DDX46 | PRPF18 | | SNRPG | | HNRNPH3 | SRSF3 |
| DDX5 | PRPF19 | | SNU13 | | HNRNPK | SRSF9 |
| DHX15 | PRPF3 | | SNW1 | | HNRNPL | SRSF5 |
| DHX16 | PRPF31 | | SRPK1 | | HNRNPM | SRSF6 |
| DHX35 | PRPF38A | | SRPK2 | | HNRNPU | RALY |
| DHX38 | PRPF39 | | SRRM1 | | HNRPDL | NCBP2 |
| DHX8 | PRPF4 | | SRRM2 | | HNRPLL | NCBP1 |
| DNAJC8 | PRPF4B | | STEEP1 | | KHDRBS1 | NAA38 |
| EFTUD2 | PRPF6 | | SUGP1 | | KHDRBS2 | NHP2L1 |
| EIF4A3 | PRPF8 | | SYF2 | | KHDRBS3 | TFIP11 |
| ESS2 | PUF60 | | TCERG1 | | KHSRP | PNN |
| FAM32A | RBM10 | | THOC1 | | MBNL1 | RNPS1 |
| FAM50A | RBM17 | | THOC2 | | NOVA1 | SAP18 |
| FAM50B | RBM22 | | THOC3 | | NOVA2 | THOC4 |
| FRA10AC1 | RBM25 | | TRIR | | PCBP1 | NCBP3 |
| FRG1 | RBM42 | | TXNL4A | | PCBP2 | THOC7 |
| GPATCH1 | RBM5 | | U2AF1 | | PTBP1 | THOC5 |
| GPKOW | RBM8A | | U2AF2 | | PTBP2 | DHX57 |
| HSPA8 | RBMX2 | | U2SURP | | QKI | RBM7 |
| HSPB1 | RNF113A | | UBL5 | | RBFOX2 | ZNF207 |
| HTATSF1 | SART1 | | USP39 | | RBM4 | CFAP20 |
| IK | SDE2 | | WBP11 | | RBMX | SRSF4 |
| ISY1 | SF1 | | WBP4 | | SFPQ | DDX42 |
| KIN | SF3A1 | | WDR83 | | SFRS1 | SEC31B |
| LSM2 | SF3A2 | | XAB2 | | SFRS11 | SRSF2 |
| PPP1CA | | HNRNPA3P14 | | HNRNPA1P73 | | HNRNPCP4 |
| DDX17 | | HNRNPA1P68 | | HNRNPCP7 | | HNRNPA1P48 |
| CCAR2 | | HNRNPA1P43 | | HNRNPA1P8 | | HNRNPA1P16 |
| NRIP2 | | HNRNPA1P54 | | HNRNPA1P9 | | HNRNPA1P19 |
| PPP1R8 | | HNRNPA1P46 | | HNRNPA1P36 | | HNRNPA1P7 |
| HNRNPR | | HNRNPA1P59 | | HNRNPA1P4 | | HNRNPA3P16 |
| HNRNPAB | | HNRNPA1P42 | | HNRNPA1P38 | | HNRNPA1P11 |
| SAP30BP | | HNRNPA1P61 | | HNRNPA1P41 | | HNRNPA1P10 |
| ARGLU1 | | HNRNPLL | | HNRNPA1P14 | | HNRNPA1P52 |
| HNRNPUL1 | | HNRNPA1P57 | | HNRNPA1P15 | | HNRNPMP2 |
| DHX9 | | HNRNPA1P66 | | HNRNPRP1 | | HNRNPDLP4 |
| CIRBP | | HNRNPKP2 | | HNRNPA1P32 | | HNRNPA3P2 |
| AGGF1 | | HNRNPDLP2 | | HNRNPA3P1 | | HNRNPA1P3 |
| PRMT5 | | HNRNPA1P39 | | HNRNPA1P33 | | HNRNPDP1 |
| PPIL4 | | HNRNPCP2 | | HNRNPA1P76 | | HNRNPA1P25 |
| WDR77 | | HNRNPA1P47 | | HNRNPA1P53 | | HNRNPH3P1 |
| MATR3 | | HNRNPA3P15 | | HNRNPRP2 | | HNRNPDLP3 |
| NSRP1 | | HNRNPA1P35 | | HNRNPA3P9 | | HNRNPDLP1 |
| HSPA1A | | HNRNPA1P51 | | HNRNPKP3 | | HNRNPA1P26 |
| DNAJC6 | | HNRNPA1P21 | | HNRNPUL2-BSCL2 | | HNRNPA1P27 |
| DDX3X | | HNRNPA1P22 | | HNRNPUL2 | | RPL36A-HNRNPH2 |
| SRSF10 | | HNRNPA3P6 | | HNRNPA1P40 | | HNRNPA1P28 |
| SMN1 | | HNRNPA3P8 | | HNRNPA1P72 | | HNRNPA3P3 |
| CLNS1A | | HNRNPKP4 | | HNRNPCP6 | | HNRNPH1P2 |
| BAG2 | | HNRNPA1P17 | | HNRNPCP8 | | HNRNPCP10 |
| RBBP6 | | HNRNPA1P23 | | HNRNPA1P60 | | SRSF11 |
| CCDC130 | | HNRNPA1P20 | | HNRNPA1P34 | | SRSF3P4 |
| CDK10 | | HNRNPA1P24 | | HNRNPABP1 | | SRSF3P5 |
| CXorf56 | | HNRNPA1P65 | | HNRNPA3P10 | | SRSF3P6 |
| JUP | | HNRNPA1P67 | | HNRNPA1P69 | | SRSF12 |
| TOE1 | | HNRNPA1P55 | | HNRNPA1P70 | | SRSF3P2 |
| TTC14 | | HNRNPA1P56 | | HNRNPA1P50 | | SRSF3P1 |
| ZCCHC10 | | HNRNPA3P13 | | HNRNPA1P30 | | SRSF8 |
| YBX3 | | HNRNPD-DT | | HNRNPA1L2 | | SRSF1P1 |
| PAXBP1 | | HNRNPDL | | HNRNPA3P5 | | SRSF6P2 |
| PABPC1 | | HNRNPKP5 | | HNRNPA1P18 | | SRSF10P1 |
| THRAP3 | | HNRNPH1P3 | | HNRNPA1P31 | | SRSF9P1 |
| RACK1 | | HNRNPA1P12 | | HNRNPA1P29 | | SRSF2P1 |
| CELF1 | | HNRNPKP1 | | HNRNPUP1 | | SRSF6P1 |
| LUC7L | | HNRNPA1P13 | | HNRNPCP1 | |  |
| HNRNPCL1 | | HNRNPA3P7 | | HNRNPA1P71 | |  |
| HNRNPCL3 | | HNRNPA1P37 | | HNRNPLP2 | |  |
| HNRNPCL2 | | HNRNPLP1 | | HNRNPA1P45 | |  |
| HNRNPCL4 | | HNRNPA1P58 | | HNRNPMP1 | |  |
| HNRNPFP1 | | HNRNPA1P1 | | HNRNPA1P74 | |  |
| HNRNPA3P12 | | HNRNPA1P2 | | HNRNPA3P11 | |  |
| HNRNPA1P63 | | HNRNPA3P4 | | HNRNPA1P44 | |  |
| HNRNPA1P6 | | HNRNPDP2 | | HNRNPA1P5 | |  |
| HNRNPCP9 | | HNRNPH1P1 | | HNRNPCP3 | |  |
| HNRNPA1P64 | | HNRNPA1P49 | | HNRNPA1P62 | |  |

**Uncropped images of western blots**


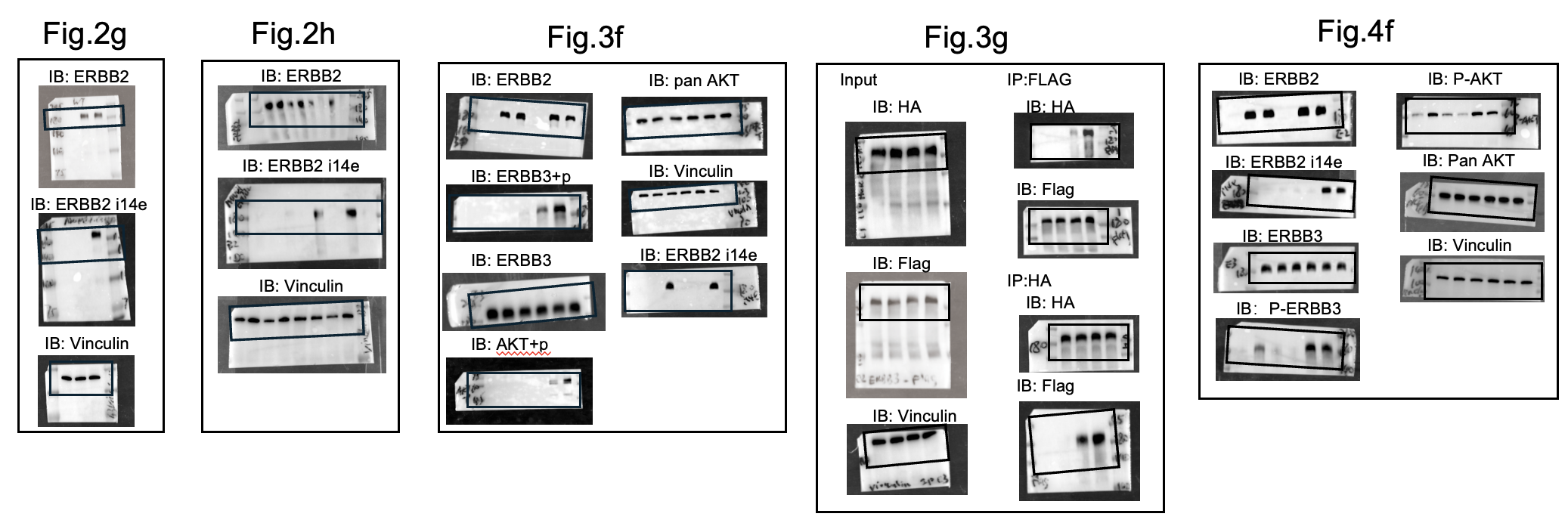


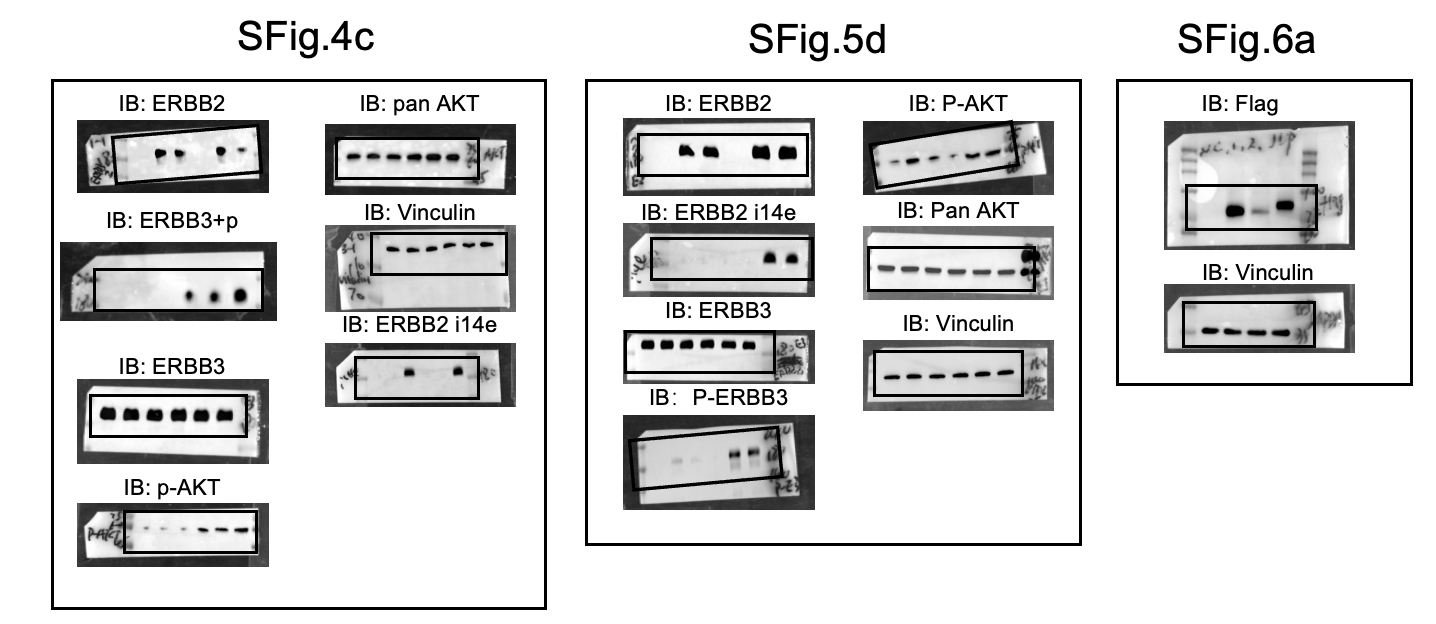


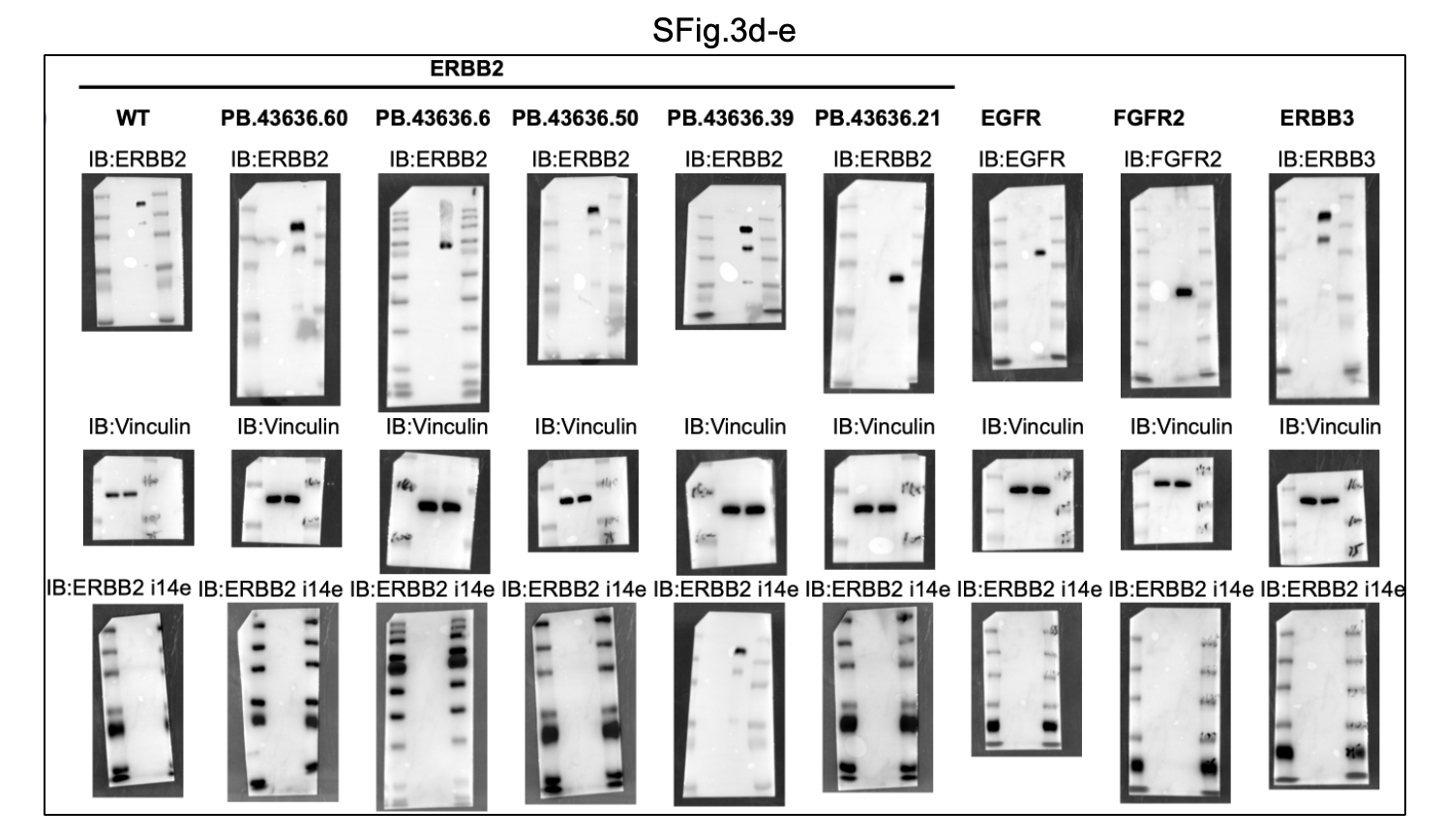

Supplement: Supplementary file 1 — Supplementary Materials [file 41392_2025_2150_MOESM1_ESM.docx]
